# Hemodynamic Response to Tracheal Intubation Using Indirect and Direct Laryngoscopes in Pediatric Patients: A Systematic Review and Network Meta-Analysis

**DOI:** 10.3390/children12060786

**Published:** 2025-06-16

**Authors:** Risa Takeuchi, Hiroshi Hoshijima, Masanori Tsukamoto, Shinichi Kokubu, Takahiro Mihara, Toshiya Shiga

**Affiliations:** 1 Bunkoukai Special Needs Dental Clinic, 2765-5 Ujiie, Sakura 329-1311, Japan; 2 Division of Dento-Oral Anesthesiology, Graduate School of Dentistry, Tohoku University, 4-1 Seiryomachi, Sendai 980-8575, Japan; 3 Systemic Management for Dentistry, Kagoshima University Hospital, 8-35-1 Sakuragaoka, Kagoshima 890-0075, Japan; tsukamoto@dent.kagoshima-u.ac.jp; 4 Department of Anesthesiology, Dokkyo Medical University, 880 Kitakobayashi, Mibu 321-0293, Japan; 5 Department of Health Data Science, Graduate School of Data Science, Yokohama City University, Yokohama 236-0004, Japan; 6 Department of Anesthesiology and Pain Medicine, International University of Health and Welfare Ichikawa Hospital, 6-1-4 Kounodai, Ichikawa 272-0827, Japan; qzx02115@nifty.com

**Keywords:** indirect laryngoscope, hemodynamic responses, pediatric, network meta-analysis

## Abstract

**Purpose:** Hemodynamic response, particularly increased heart rate (HR) and blood pressure, can occur during tracheal intubation and is an adverse event to be avoided. The aim of this study was to use a network meta-analysis (NMA) to develop a ranking of hemodynamic responses (HR and mean blood pressure, MBP) after intubation of indirect and direct laryngoscopes in pediatric patients. **Method:** Studies were eligible for inclusion if they had a prospective randomized design, compared hemodynamic response (HR and MBP) to tracheal intubation between indirect and/or direct laryngoscopes, and were conducted in pediatric patients. The pooled difference between each intubation device’s intubation time is expressed as a weighted mean difference (WMD) of a 95% confidence interval (CI). The intubation time of the device was evaluated using P-scores calculated from the network point estimates and standard errors. A random-effects model was used when pooling effect sizes. We also analyzed intubation time as a related factor to hemodynamic responses. **Results:** From the electronic databases, we selected 16 trials for review. In a Macintosh-referenced analysis, Airtraq suppressed an increase of HR and MBP during tracheal intubation in pediatric patients significantly more than a Macintosh laryngoscope. (HR; WMD = −16.7, 95%CI −22.5 to −10.9, MBP; WMD = −8.57, 95%CI −10.9 to −6.27). Airtraq also topped the HR and MBP P-score rankings. The results of this study showed similar laryngoscopes in the top five rankings of P-scores (Airtraq, Coopdech video laryngoscope, Miller, C-MAC, Wis-Hipple) for HR and intubation time. **Conclusions:** We applied a network meta-analysis to create a consistent ranking of intubation devices that prevent hemodynamic changes during tracheal intubation in pediatric patients. In this NMA, Airtraq proved to be the best laryngoscope for preventing hemodynamic responses during tracheal intubation in pediatric patients. In the analysis of intubation time, Airtraq showed the shortest intubation time.

## 1. Introduction

Hemodynamic response, in particular increased heart rate (HR) and blood pressure, can occur during tracheal intubation and is an adverse event to be avoided. Tracheal intubation stimulates the sympathetic nervous system and oropharyngeal and upper airway receptors, which increases blood catecholamine levels and leads to dramatic changes in hemodynamics [1,2]. In pediatric patients, the autonomic nervous system is immature, and when the sympathetic nervous system becomes dominant, excessive secretion of catecholamines causes severe tachycardia, arrhythmia, myocardial ischemia, and cardiac arrest [3]. Furthermore, in children, inhalational anesthetics used for induction of anesthesia, such as sevoflurane and halothane, interact with catecholamines and can cause further complications, including myocardial ischemia and severe arrhythmia [4,5].

In clinical settings, a range of indirect laryngoscopes is employed for tracheal intubation in pediatric patients. Unlike direct laryngoscopes such as Macintosh or Miller, which require alignment of the oral, pharyngeal, and laryngeal axes to obtain a direct view of the glottis, indirect laryngoscopes are equipped with a camera at the blade tip that transmits glottic images to an external screen [6,7]. This design feature eliminates the necessity of aligning the three anatomical axes, potentially making the procedure less invasive [8,9,10]. As a result, indirect laryngoscopy may reduce the hemodynamic response associated with intubation in pediatric patients.

Evidence from adult studies, including meta-analyses, has shown that indirect laryngoscopes are linked to significantly lower hemodynamic responses during intubation compared to direct methods [11,12]. However, pediatric studies offer inconsistent findings: while some suggest a blunted hemodynamic reaction with indirect devices [8,10,13], others report no notable difference between direct and indirect approaches [9,14,15,16]. Consequently, there is still no definitive consensus regarding the effect of laryngoscope type on hemodynamic response in pediatric tracheal intubation.

This study aims to clarify this issue by conducting a network meta-analysis (NMA) to compare and rank the hemodynamic responses elicited by different laryngoscope types in children undergoing intubation.

## 2. Methods

### 2.1. Protocol and Registration

The preparation of this manuscript was guided by the PRISMA-NMA (Preferred Reporting Items for Systematic Reviews and Meta-Analyses extension for Network Meta-Analyses) guidelines [17]. The study protocol was pre-registered with PROSPERO (registration number: CRD42023360282).

### 2.2. Search Strategy

A thorough search of the PubMed, EMBASE (Excerpta Medica), and Cochrane Central Register of Controlled Trials databases was conducted in March 2023. In addition to database searches, we manually reviewed the reference lists of the selected articles and relevant review papers to identify any additional eligible studies. No limitations were placed on the type of publication or language. Details of the search methodology can be found in Supplemental S1.

### 2.3. Study Selection and Data Collection

Potentially relevant articles were extracted and assessed for eligibility independently by two authors (H.H. and T.S.) working independently. If data in the extracted articles were found to be missing or inconsistent, we contacted the authors directly for clarification. We also searched the website to confirm that the protocol for each study included in the meta-analysis had been published before the research was undertaken and that the protocol was implemented as stated. Non-publication of a protocol on the website was recorded as a risk of bias.

Studies were eligible for inclusion if they had a prospective randomized design and compared hemodynamic response (HR and mean blood pressure [MBP]) to tracheal intubation between indirect and/or direct laryngoscopes for pediatric patients. We extracted data collected from the earliest time recorded after tracheal intubation onward. Studies excluded were those involving manikins, those in which tracheal intubation was performed during cardiopulmonary resuscitation, those involving adult patients, and those that involved the use of double-lumen tubes.

### 2.4. Intubation Time

We also compared the intubation time required for each type of laryngoscope to determine if it affected the hemodynamic response. Intubation time was also ranked by NMA using continuous data.

### 2.5. Risk of Bias Within Individual Studies

The risk of bias in each included study was assessed in accordance with the guidelines outlined in the Cochrane Handbook [18] (Supplemental S2). Two reviewers (H.H. and T.M.) independently conducted the evaluations to ensure objectivity.

### 2.6. Certainty of Evidence

The quality of the evidence for each estimate in the network meta-analysis was evaluated using the GRADE methodology (Grading of Recommendations, Assessment, Development and Evaluation) [19]. To facilitate this assessment, we employed the CINeMA (Confidence in Network Meta-Analysis) web-based tool, which adheres to a previously established evaluative framework [20]. The inconsistency between direct and indirect evidence was assessed using a per-design interaction random-effects model. Significant inconsistencies were suggested when *p* < 0.05 was reported in the consistency analysis or when confidence intervals excluded nulls [21].

### 2.7. Publication Bias

To explore potential small-study effects in relation to the primary outcome, we generated a comparison-adjusted funnel plot. Prior to constructing this plot, we hypothesized that more recently introduced devices might be favored in smaller trials. Consequently, laryngoscopes were ordered chronologically from the oldest to the most recent, allowing us to standardize comparisons between earlier and newer interventions and calculate differences in effect sizes accordingly.

### 2.8. Statistical Analysis

The HR and MBP differences between each device were combined and expressed as weighted mean differences (WMD) with 95% confidence intervals (CI). Also, the difference in intubation time between each device was combined and expressed as a WMD with a 95%CI. A random-effects model was used when pooling effect sizes. The ranking of HR, MBP, and intubation time for each device was determined by calculating P-scores based on the network point estimates and standard errors. The P-score serves as a frequentist alternative to the Bayesian network surface under the cumulative ranking curve and represents the average level of certainty that one intervention is superior to another. P-scores can be used to rank interventions on a scale of 0 (worst) to 1 (best) within a range of interventions. This NMA will be performed using R version 4.2.2 (R Foundation for Statistical Computing, Vienna, Austria) with the netmeta package. A random-effects model was used when pooling effect sizes.

## 3. Results

An initial search across the specified electronic databases yielded 750 potentially relevant studies. After screening titles and abstracts, 602 articles were excluded. The remaining 148 articles underwent full-text review to assess their eligibility based on the predefined inclusion and exclusion criteria. A further 132 studies were excluded that compared anesthetic agents (n = 60), used a laryngeal mask (n = 31), assessed cardiopulmonary complications as the outcome (n = 19), did not compare hemodynamic response (n = 10), nonrelevant studies (n = 8), compared intraoperative vital signs (n = 4), or used an unknown type of laryngoscope (n = 1). The remaining 16 articles met the inclusion criteria and contained the data necessary for the planned comparison (Supplemental S3) [8,9,10,13,15,16,22,23,24].

All of the trials eligible for inclusion in the meta-analysis were published between 2007 and 2022. Fourteen of the sixteen studies included patients aged 1 year or older or 12 years or younger. The remaining two studies included children up to 18 years of age. The most commonly used indirect laryngoscope was the Airtraq, followed by the McGrath, C-MAC, or Truview EVO2. Overall, the most commonly used laryngoscope was the Macintosh laryngoscope (used in 12 studies), followed by Airtraq and Miller, which were used in five studies. McGrath, C-MAC, Truview EVO2, and McCoy laryngoscopes were used in two studies each. Wis-Hipple and AirwayScope were used in one study. Fourteen of the sixteen studies reported the American Society of Anesthesiologists Physical Status classification, which was I–II. Fourteen studies were performed in patients with normal airways, and one study did not report airway status. One study included pediatric patients with difficult airway settings. The methods used for induction and management of anesthesia varied from study to study (Table 1).

### 3.1. Risk of Bias and Quality of Evidence

The risk of bias diagram is shown in Supplemental S4. The quality of the randomized controlled trials improved, and the risk of bias decreased between 2007 and 2022. Patient randomization was performed in all studies, but blinding as to the laryngoscope used was not possible in any of the studies. Whether the study evaluators were blinded was unclear in eight of the sixteen studies. Pre-registration of the study protocols tended to be more common for recent research, with many older studies not having a record of pre-registration.

### 3.2. Overall Analysis

#### 3.2.1. Heart Rate During Tracheal Intubation

**Figure 1:**
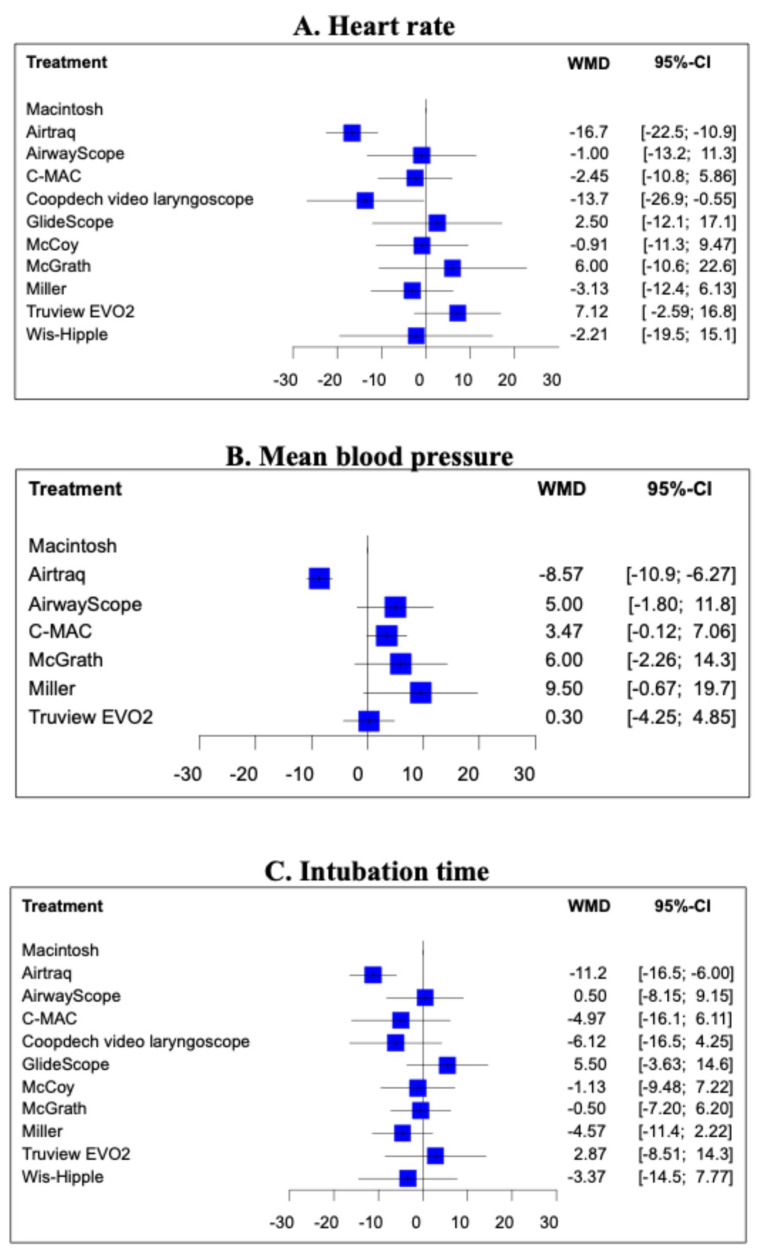
Forest plot of mean blood pressure response during tracheal intubation for each laryngoscope when referenced to Macintosh laryngoscope. A forest plot of the main results. (**A**) Forest plot of heart rate response during tracheal intubation for each laryngoscope when referenced to Macintosh laryngoscope. (**B**) Forest plot of mean blood pressure response during tracheal intubation for each laryngoscope when referenced to Macintosh laryngoscope. (**C**) Forest plot of intubation time for tracheal intubation for each laryngoscope when referenced to Macintosh laryngoscope.

**Figure 2:**
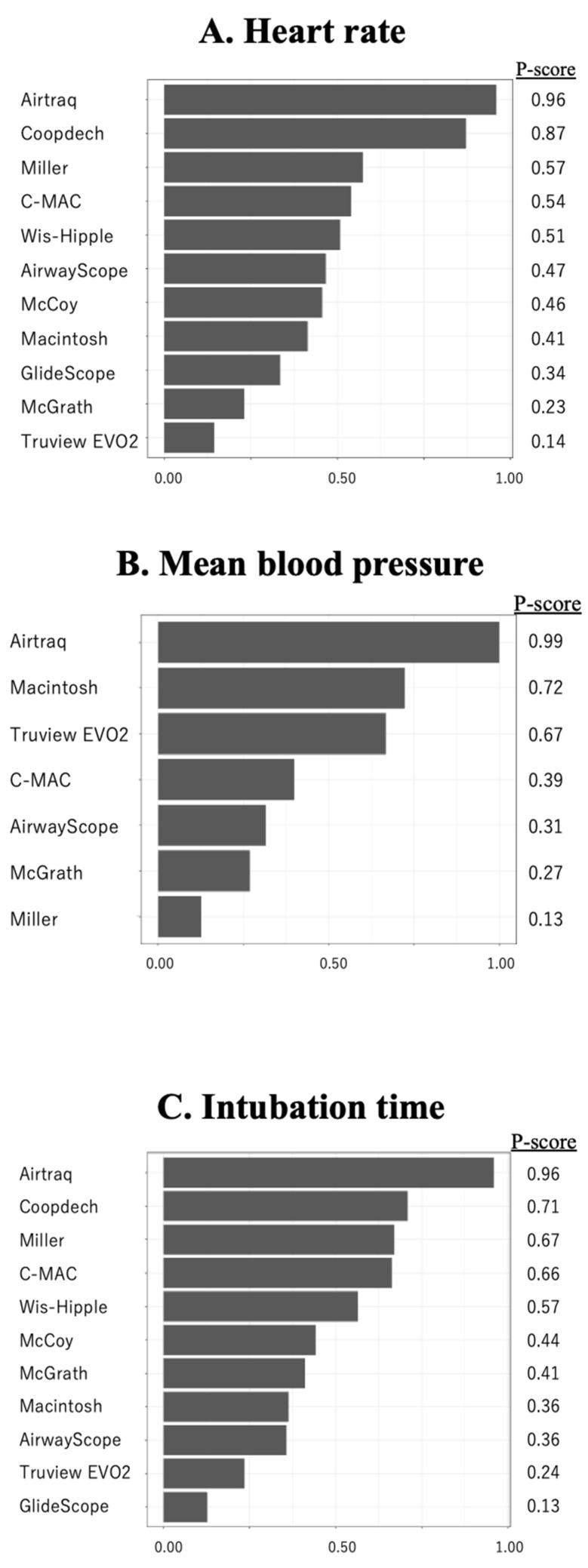
The results of heart rate, mean blood pressure, and intubation time by P score ranking. The results of heart rate (**A**), mean blood pressure (**B**), and intubation time (**C**) by P-score ranking.

Fifteen studies (1101 patients) included an analysis of HR during tracheal intubation. In a Macintosh-referenced analysis, the increase in HR during tracheal intubation in pediatric patients was suppressed to a significantly greater extent by the Airtraq and Coopdech video laryngoscopes than by the Macintosh laryngoscope (Airtraq; WMD = −16.7, 95%CI −22.5 to −10.9, Coopdech video laryngoscope; WMD = −13.7, 95%CI −26.9 to −0.55; Figure 1A). The expected P-score and ranking of each intubation device for the analysis of HR during tracheal intubation are shown in Figure 2A. The highest ranked of all the laryngoscopes was the Airtaq (P-score 0.96), while the Coopdech video laryngoscope, Miller, C-MAC, Wis-Hipple, AirwayScope, and McCoy all ranked higher than the Macintosh. The Truview EVO2 ranked lowest (P-score 0.14). A league table of the results for all comparison pairs in terms of HR is presented in Supplemental S5. Consistency analysis did not detect inconsistency across global models between the direct and indirect comparisons (*p* = 0.76). The node-splitting analysis indicated no significant discrepancies between direct and indirect comparisons (see Supplemental S6). Imprecision assessments using CiNeMA are presented in Supplemental S7. Regarding heterogeneity, 33% of the comparisons showed confidence and prediction intervals that extended toward both clinically meaningful benefit and harm, raising substantial concerns (Supplemental S8). A comparison-adjusted funnel plot is illustrated in Supplemental S9A. Egger’s test suggested the presence of publication bias in the hazard ratio (HR) data (*p* = 0.01), which are summarized in Table 2.

#### 3.2.2. Mean Blood Pressure During Tracheal Intubation

Analysis of MBP during endotracheal intubation included nine studies and 680 patients. In the Macintosh analysis, only Airtraq significantly reduced the increase in MBP during tracheal intubation compared to the Macintosh laryngoscope (WMD = −8.57, 95%CI −10.9 to −6.27; Figure 2B). The expected P-score and ranking of each intubation device with respect to MBP during tracheal intubation are shown in Figure 2B. Among all the laryngoscopes, the suppression of MBP during tracheal intubation was highest for the Airtraq (P-score 0.99) and lowest for the Miller (P-score 0.13). The Macintosh laryngoscope ranked second among all the laryngoscopes (Supplemental S10). The *p*-value for inconsistency could not be calculated because the network was star-shaped. The results of imprecision and heterogeneity obtained from CiNeMA are shown in Supplementals S11 and S12. A comparison-adjusted funnel plot is shown in Supplemental S9B. MBP had fewer than nine studies, so it was not possible to perform Egger’s test. The summary table of the MBP is shown in Table 3.

#### 3.2.3. Intubation Time

The analysis of intubation time included 13 studies and 1011 patients. In the analysis with reference to the Macintosh, only the Airtraq significantly reduced intubation time. (WMD = −12.2, 95%CI −16.5 to −6.0; Figure 1C). Figure 2C displays the anticipated P-scores and ranking for each intubation device in terms of intubation time. Among the devices, the Airtraq demonstrated the shortest intubation time, with a P-score of 0.96. The next ranked laryngoscope was the Coopdech video laryngoscope, followed by the Miller, C-MAC, and Wis-Hipple. These top-ranking results were similar to the results of the HR ranking (Supplemental S13). Consistency analysis did not detect inconsistency across global models between the direct and indirect comparisons (*p* = 0.33). The node-splitting model showed no indication of inconsistency between direct and indirect evidence. (Supplemental S14). The results of imprecision and heterogeneity obtained from CiNeMA are shown in Supplementals S15 and S16. The comparison-adjusted funnel plot and Egger’s test results indicate no publication bias (*p* = 0.11) (Supplemental S9C). The summary table of the intubation time is shown in Supplemental S17.

## 4. Discussion

In this study, we used NMA to compare hemodynamic fluctuations during tracheal intubation between direct and indirect laryngoscopes in pediatric patients. Pairwise meta-analysis showed that only the Airtraq significantly suppressed the hemodynamic response during tracheal intubation of these patients. According to the NMA P-score ranking, the Airtraq had the best ability to suppress both HR and MBP and the shortest intubation time.

The primary contributors to the hemodynamic response—such as elevated heart rate and blood pressure—observed during tracheal intubation are associated with the mechanical stimulation of the upper airway caused by laryngoscopy and tube insertion [32,33]. In direct laryngoscopy, proper visualization of the glottis typically requires alignment of the oral, pharyngeal, and tracheal axes. This process involves applying upward force with the laryngoscope blade to lift the tongue and epiglottis, which can act as a significant stimulus. Previous studies have reported that this lifting force reaches around 30–50 N when a direct laryngoscope is used [34,35,36,37].

However, there is no need to align the three axes when using an indirect laryngoscope [8,9,10], so tracheal intubation can be performed successfully even with a small lifting force [34,35]. The Airtraq, which had the highest P-score ranking in our study, has been reported to have a lifting force of only 10.4 ± 2.8 N during tracheal intubation [34]. This small lifting force is thought to have contributed to the suppression of the hemodynamic response. Similarly, the Airwayscope, which ranked high for the suppression of HR in our study, requires a median lifting force of 11 N (interquartile range, 8–14) [35]; also, the lifting force of the C-MAC laryngoscope is reported to be 11.5 ± 8.1 N [38].

Another possible explanation for the Airtraq’s ability to reduce the hemodynamic response lies in its built-in guiding channel for the endotracheal tube. This feature facilitates smoother tube advancement by helping maintain an optimal angle between the tracheal tube tip and the axis of the trachea, thereby potentially minimizing mechanical stress during intubation [39]. This reduces the stimulation of the tracheal wall by the tracheal tube and, consequently, the hemodynamic response.

Prolonged tracheal intubation increases irritation to the upper airway and heightens HR and MBP during or after tracheal intubation. In this study, we performed an NMA to determine whether the length of tracheal intubation was associated with hemodynamic responses. We found that the same laryngoscopes were in the top five P-score rankings for both HR and intubation time (i.e., the Airtraq, Coopdech video laryngoscope, Miller, C-MAC, and Wis-Hipple). Therefore, it is likely that there is some correlation between the length of tracheal intubation and fluctuations in hemodynamics in pediatric patients. However, further research is needed.

The reduction of hemodynamic responses during tracheal intubation using an intubation device has various advantages. First, it is advantageous for use in awake intubation of patients with upper airway obstruction, for whom intubation is expected to be difficult. Second, it may also be beneficial for preventing these complications in patients with potential myocardial ischemia and cerebral hemorrhage during tracheal intubation. Third, in situations where sufficient general anesthetic drugs are not available in emergency departments or ICUs, an intubation device that suppresses hemodynamic responses may provide safer intubation for these patients.

## 5. Limitations

This NMA has some potential limitations. First, it was not possible for operators to be blinded to the type of laryngoscope used, which means that the possibility of researcher bias cannot be excluded. Second, each study used various types and dosages of anesthetic agents, which may have introduced further bias because of their potential effects on the hemodynamic response during tracheal intubation. For example, some studies used glycopyrrolate, which increases heart rate (HR). Ketamine, known to increase heart rate and blood pressure, was used in some other studies. On the other hand, one of the included studies used clonidine as a premedication, which may decrease HR. Opioids such as fentanyl and sufentanil are commonly used as induction drugs and may blunt the response to intubation. The lack of information on the depth of anesthesia at the time of tracheal intubation, such as BIS or minimum alveolar concentration, creates further uncertainty about the actual cause of the hemodynamic changes observed. A third limitation is the different ages of patients in each RCT. This study included pediatric patients aged from 0 to 18 years. Since children grow in height and weight, the degree of difficulty of tracheal intubation differs depending on the target age, and the size of the laryngoscope blade used also differs. Variability in patient age further influences baseline cardiovascular tone and anesthetic sensitivity. A fourth limitation is that the hemodynamic response during tracheal intubation is only one functional aspect of laryngoscope performance and therefore must be used taking into account individual patient characteristics. Fifth, we were unable to investigate bradycardia during tracheal intubation. This NMA did not include RCTs in which bradycardia occurred during tracheal intubation, so analysis was not possible. However, bradycardia during tracheal intubation in children may be critical, so future investigation is necessary. Other limitations include differences in sample size and the skill levels of the laryngoscopists in the original studies.

## 6. Conclusions

We applied a network meta-analysis to create a consistent ranking of intubation devices that prevent hemodynamic changes during tracheal intubation in pediatric patients. In this NMA, Airtraq proved to be the best laryngoscope for preventing hemodynamic responses during tracheal intubation in pediatric patients. According to the analysis of intubation time, the Airtraq also had the shortest intubation time.

## Figures and Tables

**Table 1:** Characteristics of included studies.

Author	Year	Type of Laryngoscope	Patient Age or Body Weight	ASA Status	Airway Condition	Type of Surgery	Methods of Induction Anesthesia
RajsiShah R [25]	2022	Macintosh (40) Airtraq (40)	3–12 y	I-II	Normal	Elective surgery	Premedication: glycopyrrolate 5 μg/kg, midazolam 0.5 mg. Anesthesia induction: propofol 2 mg/kg, suxamethonium 2 mg/kg.
Hur M [26]	2021	Macintosh (15) McGrath (15)	1–10 y	I-II	Difficult (Torticollis)	Torticollis surgery	Anesthesia induction: propofol 2.5–3.0 mg/kg, fentanyl 1 mcg/kg, rocuronium 1 mg/kg.
Soltani AE [22]	2020	Macintosh (30) Miller (32)	0–4 y	I-II	Normal	Elective surgery	Premedication: midazolam 0.5 mg/kg and ketamine 2.5 mg/kg. Anesthesia induction: sevoflurane 8%, fentanyl 1 μg/kg
Elattar H [27]	2020	Miller (29) C-MAC (36) Wis-Hipple (31)	<18 y	I-II	Normal	Elective surgery	Anesthesia induction: sevoflurane 8%, nitrous oxide (66%, and oxygen 33%, (propofol 2–3 mg/kg).
Yi IK [23]	2019	Macintosh (68) AirwayScope (68)	1–10 y	I-II	Normal	Elective surgery	No premedication. Anesthesia induction: sevoflurane 5–8%, oxygen 100%, (propofol 2.5–3 mg/kg), rocuronium 0.6 mg/kg.
Pangasa N [28]	2019	Macintosh (25) Truview EVO2 (25)	2–8 y	I-II	Normal	Elective surgery	Premedication: oral midazolam 0.5 mg/kg. Anesthesia induction: sevoflurane 8%, oxygen, rocuronium 0.6 mg/kg, fentanyl 2 μgm/kg.
Yadav P [29]	2019	Macintosh (25) Miller (25) McCoy (25)	2–6 y	I-II	Normal	Elective surgery	Anesthesia induction: halothane and oxygen, fentanyl 2 μg/kg, atracurium 0.5 mg/kg, glycopyrrolate 0.01 mg/kg.
Orozco JA [10]	2018	Macintosh (40) Airtraq (40)	2–8 y	I-II	Normal	laparoscopic cholecystectomy	Premedication: clonidine 0.5 g/kg. Anesthesia induction: propofol 2.5 mg/kg, rocuronium 0.6 mg/kg, fentanyl 1.5 g/kg, 1% lidocaine.
Giraudon A [9]	2017	Macintosh (67) McGrath (65)	10–20 kg	I-II	Normal	Elective surgery	Anesthesia induction: sevoflurane (end tidal concentration of 4.5%), (unknown neuromuscular blockade), sufentanil 0.3 mcg/kg, or alfentanil 20 mg/kg.
Das B [8]	2017	Miller (30) Airtraq (30)	2–10 y	I-II	Normal	Elective surgery	Premedication: oral midazolam 0.3 mg/kg. Anesthesia induction: sevoflurane, oxygen, rocuronium 0.6 mg/kg.
Patil VV [15]	2016	Macintosh (30) C-MAC (30)	8–18 y	I-II	Normal	Tonsillectomy surgeries	Premedication: glycopyrrolate 10 μg/kg, fentanyl 2 μg/kg, midazolam 10 μg/kg intravenous. Anesthesia induction: propofol 2 mg/kg, vecuronium 0.1 mg/kg.
Riad W [13]	2012	Macintosh (25) Airtraq (25)	2–10 y	I	Normal	Elective surgery	Premedication: oral midazolam 0.5 mg/kg. Anesthesia induction: sevoflurane, oxygen, fentanyl 2 μg/kg, atracurium 0.5 mg/kg, glycopyrrolate 0.04 μg/kg.
Inal MT [16]	2010	Miller (25) Truview EVO2 (25)	2–8 y	N/A	N/A	N/A	Anesthesia induction: sevoflurane, oxygen, nitrous oxide 60%, rocuronium 0.8 mg/kg.
Shayeghi S [24]	2007	Macintosh (32) GlideScope (30)	Infant (2.56 y ± 2.19, 3.21 y ± 1.77)	N/A	Normal	Elective surgery	Anesthesia induction: thiopental sodium 6 mg/kg, atracurium 0.5 mg/kg.
Hazarika R [30]	2006	Airtraq (50) Coopdech video laryngoscope (50)	6–36 m	I-II	Normal	Elective surgery	Premedication: midazolam 0.3 mg/kg, Anesthesia induction: sevoflurane 8%, nitrous oxide (66% nitrous oxide and oxygen 33%), fentanyl 2 μg/kg, atracurium 0.5 mg/kg.
Iohom G [31]	2004	Miller (20) McCoy (20)	0–6 m	I-II	Normal	Elective surgery	Anesthesia induction: sevoflurane 8%, nitrous oxide (66% nitrous oxide and oxygen 33%).
American Society of Anesthesiologists-Physical Status classification (ASA-PS)							

**Table 2:** Summary of findings table for heart rate.

**Patients:** pediatric patients who received tracheal intubation		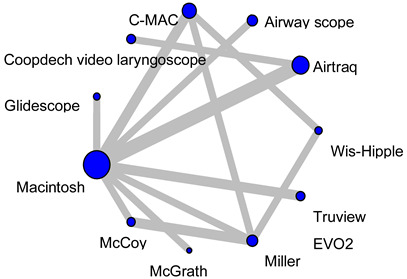		
**Interventions:** indirect laryngoscope, miller laryngoscope				
**Comparator (reference):** macintosh laryngoscope				
**Outcome:** heart rate				
**Setting:** elective surgery				
**Total studies: 15 RCT** **Total Participants: 1101**	**Mean difference** **(95%CI)**	**Certainty of evidence**	**Reasons for** **downgrading**	**P score**
Airtraq (4 RCT; 145 participants)	−16.7 (−22.5 to −10.9)	⨁⨁**◯◯** **Low**	Within-study bias and reporting bias	0.96
Airway scope (1 RCT; 68 participants)	−1.00 (−13.3 to 11.3)	⨁**◯◯◯** **Very low**	Within-study bias, reporting bias, and imprecision	0.47
C-MAC (2 RCT; 96 participants)	−2.45 (−10.8 to 5.86)	⨁**◯◯◯** **Very low**	Within-study bias, reporting bias, imprecision, and heterogeneity	0.54
Coopdech video laryngoscope (1 RCT; 50 participants)	−13.7 (−26.9 to −0.55)	⨁**◯◯◯** **Very low**	Within-study bias, reporting bias, and heterogeneity	0.87
Glidescope (1 RCT; 30 participants)	2.20 (−12.1 to 17.1)	⨁⨁**◯◯** **Low**	Within-study bias and reporting bias	0.34
Macintosh (11 RCT; 397 participants)	Not estimable	**Reference comparator**	Not estimable	0.41
McCoy (1 RCT; 45 participants)	6.00 (−10.6 to 22.6)	⨁**◯◯◯** **Very low**	Within-study bias, reporting bias, imprecision, and heterogeneity	0.46
McGrath (2 RCT; 80 participants)	−3.47 (−10.8 to −3.39)	⨁**◯◯◯** **Very low**	Within-study bias, reporting bias, and imprecision	0.23
Miller (6 RCT; 158 participants)	−3.13 (−12.4 to 6.13)	⨁**◯◯◯** **Very low**	Within-study bias, reporting bias, imprecision, and heterogeneity	0.57
Truview EVO2 (2 RCT; 50 participants)	7.12 (−2.59 to 16.8)	⨁**◯◯◯** **Very low**	Within-study bias, reporting bias, imprecision, and heterogeneity	0.14
Wis-Hipple (1 RCT; 31 participants)	−2.21 (−19.5 to 15.1)	⨁**◯◯◯** **Very low**	Within-study bias, reporting bias, and imprecision	0.51

RCT: Randomized controlled trial, CI:Confidence interval.

**Table 3:** Summary of findings table for mean blood pressure.

**Patients:** pediatric patients who received tracheal intubation		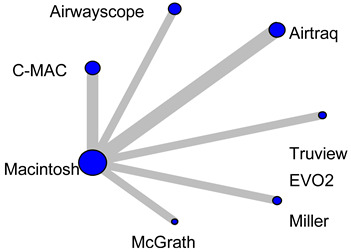		
**Interventions:** indirect laryngoscope, miller laryngoscope				
**Comparator (reference):** macintosh laryngoscope				
**Outcome:** mean blood pressure				
**Setting:** elective surgery				
**Total studies: 9 RCT** **Total Participants: 680**	**Mean diffence** **(95%CI)**	**Certainty of evidence**	**Reasons for** **downgrading**	**P score**
Airtraq (3 RCT; 110 participants)	−8.57 (−10.9 to −6.27)	⨁**◯◯◯** **Very low**	Within-study bias, reporting bias, heterogeneity, and incoherence	0.99
Airway scope (1 RCT; 68 participants)	5.00 (−1.80 to 11.8)	⨁**◯◯◯** **Very low**	Within-study bias, reporting bias, imprecision, heterogeneity, and incoherence	0.32
C-MAC (2 RCT; 95 participants)	3.74 (−0.12 to 7.06)	⨁**◯◯◯** **Very low**	Within-study bias, imprecision, heterogeneity, and incoherence	0.39
Macintosh (9 RCT; 335 participants)	Not estimable	**Reference comparator**	Not estimable	0.72
McGrath (1 RCT; 15 participants)	6.00 (−2.26 to 14.26)	⨁**◯◯◯** **Very low**	Within-study bias, reporting bias, imprecision, heterogeneity, and incoherence	0.27
Miller (1 RCT; 32 participants)	9.50 (−0.67 to 19.7)	⨁**◯◯◯** **Very low**	Within-study bias, reporting bias, imprecision, heterogeneity, and incoherence	0.13
Truview EVO2 (1 RCT; 25 participants)	0.30 (−4.25 to 4.85)	⨁**◯◯◯** **Very low**	Within-study bias, imprecision, heterogeneity, and incoherence	0.67

RCT: Randomized controlled trial, CI:Confidence interval.

## Supplementary Materials

The following supporting information can be downloaded at https://www.mdpi.com/article/10.3390/children12060786/s1: Supplemental S1: Search strategy. Supplemental S2: The methodological domain of risks of bias. Supplemental S3: Meta-analysis flow chart. RCT, randomized controlled trial. Supplemental S4: The risk of bias assessment. Green circles, red circles, and yellow circles indicate “low risk of bias”, “high risk of bias”, and “unclear risk of bias”, respectively. Supplemental S5: League table of the heart rate. Supplemental S6: Results of the inconsistency of the heart rate. Supplemental S7: Results of the imprecision of the heart rate. Supplemental S8: Results of heterogeneity of the heart rate. Supplemental S9: Results of the funnel plot of the heart rate (A), mean blood pressure (B), and intubation time (C). Supplemental S10: League table of the mean blood pressure. Supplemental 11: Results of imprecision of the mean blood pressure. Supplemental S12: Results of heterogeneity of the mean blood pressure. Supplemental S13: League table of the intubation time. Supplemental S14: Results of the inconsistency of the intubation time. Supplemental S15: Results of the imprecision of the intubation time. Supplemental S16: Results of heterogeneity of the intubation time. Supplemental S17: Summary of findings table for the intubation time.
