# Supplementary material for: Hemodynamic Response to Tracheal Intubation Using Indirect and Direct Laryngoscopes in Pediatric Patients: A Systematic Review and Network Meta-Analysis
Source: Children (Basel). 2025 Jun 16;12(6):786. doi: 10.3390/children12060786 (PMC12191819; doi:10.3390/children12060786)
Supplement: Supplementary file 1 [file children-12-00786-s001.zip › children-3685955-supplementary.pdf]

Supplemental File

Supplemental S1 Search strategy.

Search strategy for pubmed

("paediatrics"[All Fields] OR "pediatrics"[MeSH Terms] OR "pediatrics"[All Fields] OR "paediatric"[All Fields] OR "pediatric"[All Fields]) AND ("intubate"[All Fields] OR "intubated"[All Fields] OR "intubates"[All Fields] OR "intubating"[All Fields] OR "intubation"[MeSH Terms] OR "intubation"[All Fields] OR "intubations"[All Fields] OR "intubator"[All Fields] OR "intubator s"[All Fields] OR "intubators"[All Fields]) AND ("haemodynamic"[All Fields] OR "hemodynamics"[MeSH Terms] OR "hemodynamics"[All Fields] OR "hemodynamic"[All Fields] OR "haemodynamical"[All Fields] OR "haemodynamically"[All Fields] OR "haemodynamics"[All Fields] OR "hemodynamical"[All Fields] OR "hemodynamically"[All Fields]) AND ("response"[All Fields] OR "responses"[All Fields] OR "responsive"[All Fields] OR "responsiveness"[All Fields] OR "responsivenesses"[All Fields] OR "responsives"[All Fields] OR "responsivities"[All Fields] OR "responsivity"[All Fields])

Search strategy for Cochrane library

#1 intubate
#2 tracheal intubation
#3 #1 or #2
#4 pediatrics
#5 paediatrics
#6 child
#7 #4 or #5 or #6
#8 hemodynamic
#9 haemodynamic
#10 #8 or #9

Search strategy for Embase

('tracheal intubation':ab,ti OR 'pediatrics':ab,ti OR 'hemodynamic':ab,ti OR 'videolaryngoscopy':ab,ti OR 'videolaryngoscope':ab,ti OR 'video laryngoscopy':ab,ti OR 'video laryngoscope':ab,ti OR indirect laryngoscope:ab,ti OR macintosh laryngoscope:ab,ti) AND (emergen*:ab,ti,kw OR operation*:ab,kw,ti) AND ('clinical article'/de OR 'clinical trial'/de OR 'comparative study'/de OR 'controlled clinical trial'/de OR 'controlled study'/de OR 'crossover procedure'/de OR 'human'/de OR 'human

experiment'/de OR 'major clinical study'/de OR 'prospective study'/de OR 'randomized controlled trial'/de OR 'randomized controlled trial (topic)'/de)

Supplemental S2 The methodological domain of risks of bias.

The risks of bias was estimated in the following methodological domains: sequence generation; allocation concealment; blinding of participants; incomplete outcome data; selective outcome reporting; and other potential threats to validity.

Supplemental S3 Meta-analysis flow chart.

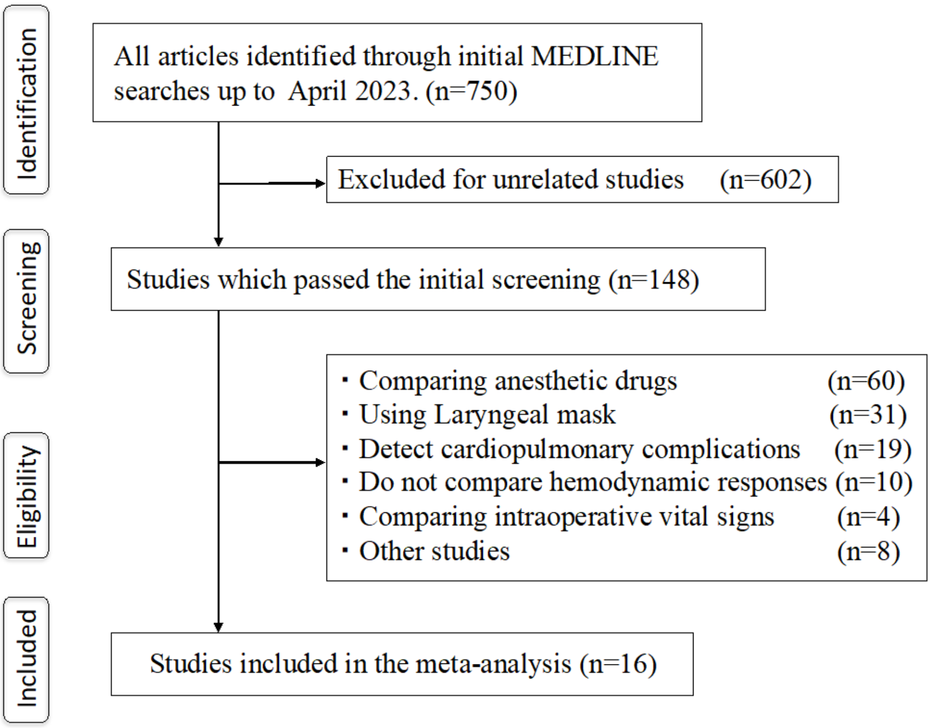

Supplemental S4 The risk of bias assessment.

Author	Year	Random sequence generation	Allocation concealment	Blind of participants and personnel	Blind of outcome assessmant	Incomplete outcome data	Selective reporting	Other potential threats to validity
Shah R	2022	+	?	−	?	+	?	?
Hur M	2021	+	+	−	+	+	+	+
Soltani AE	2020	+	+	−	?	+	?	+
Elattar H	2020	+	+	−	+	+	+	+
Yi IK	2019	+	?	−	?	+	+	+
Pangasa N	2019	+	+	−	?	+	+	+
Yadav P	2019	+	+	−	?	+	+	+
Orozco JA	2018	+	+	−	+	+	+	+
Giraudon A	2017	+	+	−	−	+	+	+
Das B	2017	+	+	−	+	+	+	+
Patil VV	2016	+	?	−	?	+	?	?
Riad W	2012	+	?	−	?	+	?	?
Inal MT	2010	+	?	−	?	+	?	?
Shayeghi S	2007	+	?	−	?	+	?	?
Hazarika R	2006	+	?	−	?	+	?	?
Oiohom G	2004	+	?	−	?	+	?	?

Supplemental S5 League table of the heart rate.

**Airtraq**	.	.	-3.0 (-14.8; 8.8)	.	-16.7 (-22.5; -10.9)	.	.	.	.	.
-15.7 (-29.3; -2.1)	**AirwayScope**	.	.	.	-1.0 (-13.3; 11.3)	.	.	.	.	.
-14.2 (-24.4; -4.1)	1.5 (-13.4; 16.3)	**C-MAC**	.	.	-3.5 (-12.9; 5.9)	.	.	3.0 (-11.1; 17.1)	.	1.0 (-16.4; 18.4)
-3.0 (-14.8; 8.8)	12.7 (-5.3; 30.7)	11.2 (-4.3; 26.8)	**Coopdech video laryngoscope**	.	.	.	.	.	.	.
-19.2 (-34.9; -3.5)	-3.5 (-22.6; 15.6)	-5.0 (-21.7; 11.8)	-16.2 (-35.8; 3.4)	**GlideScope**	2.5 (-12.1; 17.1)	.	.	.	.	.
-16.7 (-22.5; -10.9)	-1.0 (-13.3; 11.3)	-2.5 (-10.8; 5.9)	-13.7 (-26.9; -0.5)	2.5 (-12.1; 17.1)	**Macintosh**	1.0 (-10.5; 12.5)	-6.0 (-22.6; 10.6)	0.7 (-10.8; 12.2)	-7.1 (-16.8; 2.6)	.
-15.8 (-27.7; -3.9)	-0.1 (-16.2; 16.0)	-1.5 (-13.5; 10.4)	-12.8 (-29.5; 4.0)	3.4 (-14.5; 21.3)	0.9 (-9.5; 11.3)	**McCoy**	.	1.7 (-7.8; 11.2)	.	.
-22.7 (-40.3; -5.1)	-7.0 (-27.7; 13.7)	-8.5 (-27.1; 10.1)	-19.7 (-40.9; 1.5)	-3.5 (-25.6; 18.6)	-6.0 (-22.6; 10.6)	-6.9 (-26.5; 12.7)	**McGrath**	.	.	.
-13.6 (-24.5; -2.6)	2.1 (-13.2; 17.5)	0.7 (-9.4; 10.8)	-10.6 (-26.7; 5.5)	5.6 (-11.7; 22.9)	3.1 (-6.1; 12.4)	2.2 (-7.0; 11.4)	9.1 (-9.9; 28.2)	**Miller**	.	-2.0 (-19.0; 15.0)
-23.8 (-35.1; -12.5)	-8.1 (-23.8; 7.5)	-9.6 (-22.4; 3.2)	-20.8 (-37.2; -4.5)	-4.6 (-22.2; 12.9)	-7.1 (-16.8; 2.6)	-8.0 (-22.2; 6.2)	-1.1 (-20.4; 18.1)	-10.2 (-23.7; 3.2)	**Truview EVO2**	.
-14.5 (-32.7; 3.7)	1.2 (-20.0; 22.4)	-0.2 (-16.8; 16.3)	-11.5 (-33.2; 10.2)	4.7 (-17.9; 27.3)	2.2 (-15.0; 19.5)	1.3 (-16.9; 19.5)	8.2 (-15.8; 32.2)	-0.9 (-17.3; 15.4)	9.3 (-10.5; 29.1)	**Wis-Hipple**

Supplemental S6 Results of inconsistency of the heart rate.

Comparison	P value
Miller vs Macintosh	0.49
Miller vs Wis-Hipple	0.64
C-MAC vs Macintosh	0.64
McCoy vs Miller	0.64
C-MAC vs Wis-Hipple	0.64
C-MAC vs Miller	0.64
McCoy vs Macintosh	0.97

Supplemental S7 Results of imprecision of the heart rate.

Comparison Airtraq:Coopdech video laryngoscope
Evidence: mixed
NMA estimate: **-3.000**
95% Confidence interval: **(-14.799,8.799)**
Confidence interval extends into clinically important effects
Imprecision judgment: Some concerns

Comparison Airtraq:Macintosh
Evidence: mixed
NMA estimate: **-16.701**
95% Confidence interval: **(-22.518,-10.885)**
Confidence interval does not cross clinically important effect
Imprecision judgment: No concerns

Comparison AirwayScope:Macintosh
Evidence: mixed
NMA estimate: **-1.000**
95% Confidence interval: **(-13.270,11.270)**
Confidence interval extends into clinically important effects in both directions
Imprecision judgment: Major concerns

Comparison C-MAC:Macintosh
Evidence: mixed
NMA estimate: **-2.453**
95% Confidence interval: **(-10.764,5.858)**
Confidence interval extends into clinically important effects
Imprecision judgment: Some concerns

Comparison C-MAC:Miller
Evidence: mixed
NMA estimate: **0.672**
95% Confidence interval: **(-9.435,10.779)**
Confidence interval extends into clinically important effects
Imprecision judgment: Some concerns

Comparison C-MAC:Wis-Hipple
Evidence: mixed
NMA estimate: **-0.241**
95% Confidence interval: **(-16.808,16.325)**
Confidence interval extends into clinically important effects in both directions
Imprecision judgment: Major concerns

Comparison GlideScope:Macintosh
Evidence: mixed
NMA estimate: **2.500**
95% Confidence interval: **(-12.095,17.095)**
Confidence interval extends into clinically important effects in both directions
Imprecision judgment: Major concerns

Comparison Macintosh:McCoy
Evidence: mixed
NMA estimate: **0.905**
95% Confidence interval: **(-9.465,11.276)**
Confidence interval extends into clinically important effects
Imprecision judgment: Some concerns

Comparison Macintosh:McGrath
Evidence: mixed
NMA estimate: **-6.000**
95% Confidence interval: **(-22.639,10.639)**
Confidence interval extends into clinically important effects in both directions
Imprecision judgment: Major concerns

Comparison Macintosh:Miller
Evidence: mixed
NMA estimate: **3.125**
95% Confidence interval: **(-6.128,12.378)**
Confidence interval extends into clinically important effects
Imprecision judgment: Some concerns

Comparison Macintosh:Truview EVO2
Evidence: mixed
NMA estimate: **-7.122**
95% Confidence interval: **(-16.834,2.591)**
Confidence interval extends into clinically important effects
Imprecision judgment: Some concerns

Comparison McCoy:Miller
Evidence: mixed
NMA estimate: **2.220**
95% Confidence interval: **(-6.976,11.415)**
Confidence interval extends into clinically important effects
Imprecision judgment: Some concerns

Comparison Miller:Wis-Hipple
Evidence: mixed
NMA estimate: **-0.914**
95% Confidence interval: **(-17.273,15.445)**
Confidence interval extends into clinically important effects in both directions
Imprecision judgment: Major concerns

Comparison Airtraq:AirwayScope
Evidence: indirect
NMA estimate: **-15.701**
95% Confidence interval: **(-29.280,-2.123)**
Confidence interval does not cross clinically important effect
Imprecision judgment: No concerns

Comparison Airtraq:C-MAC
Evidence: indirect
NMA estimate: **-14.248**
95% Confidence interval: **(-24.393,-4.104)**
Confidence interval does not cross clinically important effect
Imprecision judgment: No concerns

Comparison Airtraq:GlideScope
Evidence: indirect

NMA estimate: **-19.201**
95% Confidence interval:
Confidence interval **(-34.912,-3.490)** *does not cross clinically important effect*

Imprecision judgment
No concerns

Comparison Airtraq:McCoy
Evidence: indirect

NMA estimate: **-15.796**
95% Confidence interval:
Confidence interval **(-27.687,-3.905)** *does not cross clinically important effect*

Imprecision judgment
No concerns

Comparison Airtraq:McGrath
Evidence: indirect

NMA estimate: **-22.701**
95% Confidence interval:
Confidence interval **(-40.327,-5.075)** *does not cross clinically important effect*

Imprecision judgment
No concerns

Comparison Airtraq:Miller
Evidence: indirect

NMA estimate: **-13.576**
95% Confidence interval:
Confidence interval **(-24.505,-2.647)** *does not cross clinically important effect*

Imprecision judgment
No concerns

Comparison Airtraq:Truview EVO2
Evidence: indirect

NMA estimate: **-23.823**
95% Confidence interval:
Confidence **(-35.144,-12.502)** *interval does not cross clinically important effect*

Imprecision judgment
No concerns

Comparison Airtraq:Wis-Hipple
Evidence: indirect

NMA estimate: **-14.490**
95% Confidence interval:
Confidence interval **(-32.702,3.722)** *extends into clinically important effects*

Imprecision judgment
Some concerns

Comparison AirwayScope:C-MAC
Evidence: indirect

NMA estimate: **1.453**
95% Confidence interval:
Confidence interval **(-13.367,16.273)** *extends into clinically important effects in both directions*

Imprecision judgment
Major concerns

Comparison AirwayScope:Coopdech video laryngoscope
Evidence: indirect

NMA estimate: **12.701**
95% Confidence interval:
Confidence interval **(-5.287,30.690)** *extends into clinically important effects*

Imprecision judgment
Some concerns

Comparison AirwayScope:GlideScope
Evidence: indirect

NMA estimate: **-3.500**
95% Confidence interval:
Confidence interval **(-22.567,15.567)** *extends into clinically important effects in both directions*

Imprecision judgment
Major concerns

Comparison AirwayScope:McCoy
Evidence: indirect

NMA estimate: **-0.095**
95% Confidence interval:
Confidence interval **(-16.160,15.971)** *extends into clinically important effects in both directions*

Imprecision judgment
Major concerns

Comparison AirwayScope:McGrath
Evidence: indirect

NMA estimate: **-7.000**
95% Confidence interval:
Confidence interval **(-27.674,13.674)** *extends into clinically important effects in both directions*

Imprecision judgment
Major concerns

Comparison AirwayScope:Miller
Evidence: indirect

NMA estimate: **2.125**
95% Confidence interval:
Confidence interval **(-13.242,17.493)** *extends into clinically important effects in both directions*

Imprecision judgment
Major concerns

Comparison **AirwayScope:Truview EVO2**
Evidence: indirect
NMA estimate: **-8.121**
95% Confidence interval:
Confidence interval **(-23.770,7.527)** *extends into clinically important effects*
Imprecision judgment
Some concerns

Comparison
Evidence: AirwayScope:Wis-Hipple indirect
NMA estimate: **1.212**
95% Confidence interval:
Confidence interval **(-19.964,22.387)** *extends into clinically important effects in both directions*
Imprecision judgment
Major concerns

Comparison
C-MAC:Coopdech video laryngoscope
Evidence: indirect
NMA estimate: **11.248**
95% Confidence interval:
Confidence interval **(-4.312,26.809)** *extends into clinically important effects*
Imprecision judgment
Some concerns

Comparison **C-MAC:GlideScope**
Evidence: indirect
NMA estimate: **-4.953**
95% Confidence interval:
Confidence interval **(-21.748,11.842)** *extends into clinically important effects in both directions*
Imprecision judgment
Major concerns

Comparison **C-MAC:McCoy**
Evidence: indirect
NMA estimate: **-1.548**
95% Confidence interval:
Confidence interval **(-13.519,10.423)** *extends into clinically important effects in both directions*
Imprecision judgment
Major concerns

Comparison **C-MAC:McGrath**
Evidence: indirect
NMA estimate: **-8.453**
95% Confidence interval:
Confidence interval **(-27.052,10.146)** *extends into clinically important effects in both directions*
Imprecision judgment
Major concerns

Comparison **C-MAC:Truview EVO2**
Evidence: indirect
NMA estimate: **-9.575**
95% Confidence interval:
Confidence interval **(-22.358,3.209)** *extends into clinically important effects*
Imprecision judgment
Some concerns

Comparison
Coopdech video laryngoscope:GlideScope
Evidence: indirect
NMA estimate: **-16.201**
95% Confidence interval:
Confidence interval **(-35.849,3.447)** *extends into clinically important effects*
Imprecision judgment
Some concerns

Comparison
Coopdech video laryngoscope:Macintosh
Evidence: indirect
NMA estimate: **-13.701**
95% Confidence interval:
Confidence interval **(-26.856,-0.547)** *does not cross clinically important effect*
Imprecision judgment
No concerns

Comparison
Coopdech video laryngoscope:McCoy
Evidence: indirect
NMA estimate: **-12.796**
95% Confidence interval:
Confidence interval **(-29.547,3.955)** *extends into clinically important effects*
Imprecision judgment
Some concerns

Comparison
Coopdech video laryngoscope:McGrath
Evidence: indirect
NMA estimate: **-19.701**
95% Confidence interval:
Confidence interval **(-40.912,1.509)** *extends into clinically important effects*
Imprecision judgment
Some concerns

Comparison
Coopdech video laryngoscope:Miller
Evidence: indirect
NMA estimate: **-10.576**
95% Confidence interval:
Confidence interval **(-26.659,5.507)** *extends into clinically important effects*
Imprecision judgment
Some concerns

Comparison Coopdech video laryngoscope:Truview EVO2
Evidence: indirect

NMA estimate: **-20.823**
95% Confidence interval:
Confidence interval **(-37.175,-4.471)** *does not cross clinically important effect*

Imprecision judgment
No concerns

Comparison Coopdech video laryngoscope:Wis-Hipple
Evidence: indirect

NMA estimate: **-11.490**
95% Confidence interval:
Confidence interval **(-33.190,10.210)** *extends into clinically important effects in both directions*

Imprecision judgment
Major concerns

Comparison GlideScope:McCoy
Evidence: indirect

NMA estimate: **3.405**
95% Confidence interval:
Confidence interval **(-14.499,21.309)** *extends into clinically important effects in both directions*

Imprecision judgment
Major concerns

Comparison GlideScope:McGrath
Evidence: indirect

NMA estimate: **-3.500**
95% Confidence interval:
Confidence interval **(-25.632,18.632)** *extends into clinically important effects in both directions*

Imprecision judgment
Major concerns

Comparison GlideScope:Miller
Evidence: indirect

NMA estimate: **5.625**
95% Confidence interval:
Confidence interval **(-11.655,22.906)** *extends into clinically important effects in both directions*

Imprecision judgment
Major concerns

Comparison GlideScope:Truview EVO2
Evidence: indirect

NMA estimate: **-4.622**
95% Confidence interval:
Confidence interval **(-22.152,12.909)** *extends into clinically important effects in both directions*

Imprecision judgment
Major concerns

Comparison GlideScope:Wis-Hipple
Evidence: indirect

NMA estimate: **4.712**
95% Confidence interval:
Confidence interval **(-17.891,27.314)** *extends into clinically important effects in both directions*

Imprecision judgment
Major concerns

Comparison Macintosh:Wis-Hipple
Evidence: indirect

NMA estimate: **2.212**
95% Confidence interval:
Confidence interval **(-15.047,19.470)** *extends into clinically important effects in both directions*

Imprecision judgment
Major concerns

Comparison McCoy:McGrath
Evidence: indirect

NMA estimate: **-6.905**
95% Confidence interval:
Confidence interval **(-26.511,12.701)** *extends into clinically important effects in both directions*

Imprecision judgment
Major concerns

Comparison McCoy:Truview EVO2
Evidence: indirect

NMA estimate: **-8.027**
95% Confidence interval:
Confidence interval **(-22.235,6.182)** *extends into clinically important effects*

Imprecision judgment
Some concerns

Comparison McCoy:Wis-Hipple
Evidence: indirect

NMA estimate: **1.306**
95% Confidence interval:
Confidence interval **(-16.913,19.525)** *extends into clinically important effects in both directions*

Imprecision judgment
Major concerns

Comparison McGrath:Miller
Evidence: indirect

NMA estimate: **9.125**
95% Confidence interval:
Confidence interval **(-9.913,28.164)** *extends into clinically important effects*

Imprecision judgment
Some concerns

Comparison McGrath:Truview EVO2
Evidence: indirect

NMA estimate: **-1.121**

95% Confidence interval: **(-20.387,18.144)**

Confidence interval extends into clinically important effects in both directions

Imprecision judgment

Major concerns

Comparison McGrath:Wis-Hipple
Evidence: indirect

NMA estimate: **8.211**

95% Confidence interval: **(-15.761,32.184)**

Confidence interval extends into clinically important effects in both directions

Imprecision judgment

Major concerns

Comparison Miller:Truview EVO2
Evidence: indirect

NMA estimate: **-10.247**

95% Confidence interval: **(-23.661,3.167)**

Confidence interval extends into clinically important effects

Imprecision judgment

Some concerns

Comparison Truview EVO2:Wis-Hipple
Evidence: indirect

NMA estimate: **9.333**

95% Confidence interval: **(-10.471,29.137)**

Confidence interval extends into clinically important effects in both directions

Imprecision judgment

Major concerns

Supplemental S8 Results of heterogeneity of the heart rate.

Comparison	Evidence	NMA estimate	95% intervals for NMA estimate: Confidence interval	95% intervals for NMA estimate: Prediction interval		Heterogeneity judgment
Airtraq:Coopdech video laryngoscope	mixed	-3.000	(-14.799,8.799)	(-23.361,17.361)	*Prediction interval extends into clinically important or unimportant effects*	Some concerns
Airtraq:Macintosh	mixed	-16.701	(-22.518,-10.885)	(-32.523,-0.880)	*Confidence and prediction intervals agree in relation to clinically important effect*	No concerns
AirwayScope:Macintosh	mixed	-1.000	(-13.270,11.270)	(-21.790,19.790)	*Confidence and prediction intervals agree in relation to clinically important effect*	No concerns
C-MAC:Macintosh	mixed	-2.453	(-10.764,5.858)	(-19.924,15.018)	*Prediction interval extends into clinically important or unimportant effects*	Some concerns
C-MAC:Miller	mixed	0.672	(-9.435,10.779)	(-18.217,19.561)	*Prediction interval extends into clinically important or unimportant effects*	Some concerns
C-MAC:Wis-Hipple	mixed	-0.241	(-16.808,16.325)	(-25.248,24.765)	*Confidence and prediction intervals agree in relation to clinically important effect*	No concerns
GlideScope:Macintosh	mixed	2.500	(-12.095,17.095)	(-20.512,25.512)	*Confidence and prediction intervals agree in relation to clinically important effect*	No concerns
Macintosh:McCoy	mixed	0.905	(-9.465,11.276)	(-18.205,20.016)	*Prediction interval extends into clinically important or unimportant effects*	Some concerns
Macintosh:McGrath	mixed	-6.000	(-22.639,10.639)	(-31.081,19.081)	*Confidence and prediction intervals agree in relation to clinically important effect*	No concerns

Comparison Macintosh:Miller
Evidence: mixed

NMA estimate: **3.125**
95% intervals for NMA estimate
Confidence interval: **(-6.128,12.378)**
Prediction interval: **(-15.069,21.319)**

Prediction interval extends into clinically important or unimportant effects

Heterogeneity judgment
Some concerns

Comparison
Evidence: Macintosh:Truview EVO2 mixed

NMA estimate: **-7.122**
95% intervals for NMA estimate
Confidence interval: **(-16.834,2.591)**
Prediction interval: **(-25.685,11.442)**

Prediction interval extends into clinically important or unimportant effects

Heterogeneity judgment
Some concerns

Comparison McCoy:Miller
Evidence: mixed

NMA estimate: **2.220**
95% intervals for NMA estimate
Confidence interval: **(-6.976,11.415)**
Prediction interval: **(-15.929,20.369)**

Prediction interval extends into clinically important or unimportant effects

Heterogeneity judgment
Some concerns

Comparison Miller:Wis-Hipple
Evidence: mixed

NMA estimate: **-0.914**
95% intervals for NMA estimate
Confidence interval:
Prediction interval: **(-17.273,15.445)**
Confidence and **(-25.707,23.879)**
prediction intervals agree in relation to clinically important effect

Heterogeneity judgment
No concerns

Comparison Airtraq:AirwayScope
Evidence: indirect

NMA estimate: **-15.701**
95% intervals for NMA estimate
Confidence interval:**(-29.280,-2.123)**
Prediction interval: **(-37.723,6.321)**

Prediction interval extends into clinically important or unimportant effects

Heterogeneity judgment
Some concerns

Comparison Airtraq:C-MAC
Evidence: indirect

NMA estimate: **-14.248**
95% intervals for NMA estimate
Confidence interval:**(-24.393,-4.104)**
Prediction interval: **(-33.169,4.672)**

Prediction interval extends into clinically important or unimportant effects

Heterogeneity judgment
Some concerns

Comparison Airtraq:GlideScope
Evidence: indirect

NMA estimate: **-19.201**
95% intervals for NMA estimate
Confidence interval:**(-34.912,-3.490)**
Prediction interval: **(-43.332,4.930)**

Prediction interval extends into clinically important or unimportant effects

Heterogeneity judgment
Some concerns

Comparison Airtraq:McCoy
Evidence: indirect

NMA estimate: **-15.796**
95% intervals for NMA estimate
Confidence interval:**(-27.687,-3.905)**
Prediction interval: **(-36.240,4.648)**

Prediction interval extends into clinically important or unimportant effects

Heterogeneity judgment
Some concerns

Comparison Airtraq:McGrath
Evidence: indirect

NMA estimate: **-22.701**
95% intervals for NMA estimate
Confidence interval:**(-40.327,-5.075)**
Prediction interval: **(-48.813,3.410)**

Prediction interval extends into clinically important or unimportant effects

Heterogeneity judgment
Some concerns

Comparison Airtraq:Miller
Evidence: indirect

NMA estimate: **-13.576**
95% intervals for NMA estimate
Confidence interval:**(-24.505,-2.647)**
Prediction interval: **(-33.166,6.014)**

Prediction interval extends into clinically important or unimportant effects

Heterogeneity judgment
Some concerns

Comparison Airtraq:Truview EVO2
Evidence: indirect

NMA estimate: **-23.823**
95% intervals for NMA estimate
Confidence interval:
Prediction **(-35.144,-12.502)**
interval: **(-43.756,-3.889)**

Confidence and prediction intervals agree in relation to clinically important effect

Heterogeneity judgment
No concerns

Comparison Airtraq:Wis-Hipple
Evidence: indirect

NMA estimate: **-14.490**
95% intervals for NMA estimate
Confidence interval: **(-32.702,3.722)**
Prediction interval: **(-41.221,12.241)**

Prediction interval extends into clinically important or unimportant effects

Heterogeneity judgment
Some concerns

Comparison **AirwayScope:C-MAC**
Evidence: indirect

NMA estimate: **1.453**
95% intervals for NMA estimate
Confidence interval:
Prediction interval: **(-13.367,16.273)**
Confidence and **(-21.783,24.689)**
prediction intervals agree in relation to clinically important effect

Heterogeneity judgment
No concerns

Comparison
AirwayScope:Coopdech video laryngoscope
Evidence: indirect

NMA estimate: **12.701**
95% intervals for NMA estimate
Confidence interval: **(-5.287,30.690)**
Prediction interval: **(-13.793,39.196)**
Prediction interval extends into clinically important or unimportant effects

Heterogeneity judgment
Some concerns

Comparison
Evidence: AirwayScope:GlideScope indirect

NMA estimate: **-3.500**
95% intervals for NMA estimate
Confidence interval:
Prediction interval: **(-22.567,15.567)**
Confidence and **(-31.145,24.145)**
prediction intervals agree in relation to clinically important effect

Heterogeneity judgment
No concerns

Comparison **AirwayScope:McCoy**
Evidence: indirect

NMA estimate: **-0.095**
95% intervals for NMA estimate
Confidence interval:
Prediction interval: **(-16.160,15.971)**
Confidence and **(-24.587,24.398)**
prediction intervals agree in relation to clinically important effect

Heterogeneity judgment
No concerns

Comparison **AirwayScope:McGrath**
Evidence: indirect

NMA estimate: **-7.000**
95% intervals for NMA estimate
Confidence interval:
Prediction interval: **(-27.674,13.674)**
Confidence and **(-36.389,22.389)**
prediction intervals agree in relation to clinically important effect

Heterogeneity judgment
No concerns

Comparison **AirwayScope:Miller**
Evidence: indirect

NMA estimate: **2.125**
95% intervals for NMA estimate
Confidence interval:
Prediction interval: **(-13.242,17.493)**
Confidence and **(-21.659,25.909)**
prediction intervals agree in relation to clinically important effect

Heterogeneity judgment
No concerns

Comparison
AirwayScope:Truview EVO2
Evidence: indirect

NMA estimate: **-8.121**
95% intervals for NMA estimate
Confidence interval: **(-23.770,7.527)**
Prediction interval: **(-32.189,15.946)**
Prediction interval extends into clinically important or unimportant effects

Heterogeneity judgment
Some concerns

Comparison
Evidence: AirwayScope:Wis-Hipple indirect

NMA estimate: **1.212**
95% intervals for NMA estimate
Confidence interval:
Prediction interval: **(-19.964,22.387)**
Confidence and **(-28.730,31.153)**
prediction intervals agree in relation to clinically important effect

Heterogeneity judgment
No concerns

Comparison
C-MAC:Coopdech video laryngoscope
Evidence: indirect

NMA estimate: **11.248**
95% intervals for NMA estimate
Confidence interval: **(-4.312,26.809)**
Prediction interval: **(-12.730,35.227)**
Prediction interval extends into clinically important or unimportant effects

Heterogeneity judgment
Some concerns

Comparison **C-MAC:GlideScope**
Evidence: indirect

NMA estimate: **-4.953**
95% intervals for NMA estimate
Confidence interval:
Prediction interval: **(-21.748,11.842)**
Confidence and **(-30.197,20.291)**
prediction intervals agree in relation to clinically important effect

Heterogeneity judgment
No concerns

Comparison **C-MAC:McCoy**
Evidence: indirect

NMA estimate: **-1.548**
95% intervals for NMA estimate
Confidence interval:
Prediction interval: **(-13.519,10.423)**
Confidence and **(-22.064,18.969)**
prediction intervals agree in relation to clinically important effect

Heterogeneity judgment
No concerns

Comparison **C-MAC:McGrath**
Evidence: indirect

NMA estimate: **-8.453**
95% intervals for NMA estimate
Confidence interval:
Prediction interval: **(-27.052,10.146)**
Confidence and **(-35.596,18.690)**
prediction intervals agree in relation to clinically important effect

Heterogeneity judgment
No concerns

Comparison **C-MAC:Truview EVO2**
Evidence: indirect

NMA estimate: **-9.575**
95% intervals for NMA estimate
Confidence interval: **(-22.358,3.209)**
Prediction interval: **(-30.841,11.692)**

Prediction interval extends into clinically important or unimportant effects

Heterogeneity judgment
Some concerns

Comparison
Coopdech video laryngoscope:GlideScope
Evidence: indirect

NMA estimate: **-16.201**
95% intervals for NMA estimate
Confidence interval: **(-35.849,3.447)**
Prediction interval: **(-44.473,12.070)**

Prediction interval extends into clinically important or unimportant effects

Heterogeneity judgment
Some concerns

Comparison
Coopdech video laryngoscope:Macintosh
Evidence: indirect

NMA estimate: **-13.701**
95% intervals for NMA estimate
Confidence interval:**(-26.856,-0.547)**
Prediction interval: **(-35.318,7.916)**

Prediction interval extends into clinically important or unimportant effects

Heterogeneity judgment
Some concerns

Comparison
Coopdech video laryngoscope:McCoy
Evidence: indirect

NMA estimate: **-12.796**
95% intervals for NMA estimate
Confidence interval: **(-29.547,3.955)**
Prediction interval: **(-37.994,12.402)**

Prediction interval extends into clinically important or unimportant effects

Heterogeneity judgment
Some concerns

Comparison
Coopdech video laryngoscope:McGrath
Evidence: indirect

NMA estimate: **-19.701**
95% intervals for NMA estimate
Confidence interval: **(-40.912,1.509)**
Prediction interval: **(-49.681,10.278)**

Prediction interval extends into clinically important or unimportant effects

Heterogeneity judgment
Some concerns

Comparison
Coopdech video laryngoscope:Miller
Evidence: indirect

NMA estimate: **-10.576**
95% intervals for NMA estimate
Confidence interval: **(-26.659,5.507)**
Prediction interval: **(-35.086,13.934)**

Prediction interval extends into clinically important or unimportant effects

Heterogeneity judgment
Some concerns

Comparison
Coopdech video laryngoscope:Truview EVO2
Evidence: indirect

NMA estimate: **-20.823**
95% intervals for NMA estimate
Confidence interval:**(-37.175,-4.471)**
Prediction interval: **(-45.608,3.963)**

Prediction interval extends into clinically important or unimportant effects

Heterogeneity judgment
Some concerns

Comparison
Coopdech video laryngoscope:Wis-Hipple
Evidence: indirect

NMA estimate: **-11.490**
95% intervals for NMA estimate
Confidence interval:
Prediction interval: **(-33.190,10.210)**
(-42.011,19.031)

Confidence and prediction intervals agree in relation to clinically important effect

Heterogeneity judgment
No concerns

Comparison **GlideScope:McCoy**
Evidence: indirect

NMA estimate: **3.405**
95% intervals for NMA estimate
Confidence interval:
Prediction interval: **(-14.499,21.309)**
(-22.999,29.810)

Confidence and prediction intervals agree in relation to clinically important effect

Heterogeneity judgment
No concerns

Comparison **GlideScope:McGrath**
Evidence: indirect

NMA estimate: **-3.500**
95% intervals for NMA estimate
Confidence interval:
Prediction interval: **(-25.632,18.632)**
(-34.501,27.501)

Confidence and prediction intervals agree in relation to clinically important effect

Heterogeneity judgment
No concerns

Comparison **GlideScope:Miller**
Evidence: indirect

NMA estimate: **5.625**
95% intervals for NMA estimate
Confidence interval:
Prediction interval: **(-11.655,22.906)**
(-20.124,31.374)

Confidence and prediction intervals agree in relation to clinically important effect

Heterogeneity judgment
No concerns

Comparison
GlideScope:Truview EVO2
Evidence: indirect

NMA estimate: **-4.622**
95% intervals for NMA estimate
Confidence interval:
Prediction interval: **(-22.152,12.909)**
(-30.633,21.390)

Confidence and prediction intervals agree in relation to clinically important effect

Heterogeneity judgment
No concerns

Comparison GlideScope:Wis-Hipple
Evidence: indirect

NMA estimate: **4.712**
95% intervals for NMA estimate
Confidence interval:
Prediction interval: **(-17.891,27.314)**
Confidence and **(-26.813,36.236)**
prediction intervals agree in relation to clinically important effect

Heterogeneity judgment
No concerns

Comparison Macintosh:Wis-Hipple
Evidence: indirect

NMA estimate: **2.212**
95% intervals for NMA estimate
Confidence interval:
Prediction interval: **(-15.047,19.470)**
Confidence and **(-23.515,27.937)**
prediction intervals agree in relation to clinically important effect

Heterogeneity judgment
No concerns

Comparison McCoy:McGrath
Evidence: indirect

NMA estimate: **-6.905**
95% intervals for NMA estimate
Confidence interval:
Prediction interval: **(-26.511,12.701)**
Confidence and **(-35.131,21.321)**
prediction intervals agree in relation to clinically important effect

Heterogeneity judgment
No concerns

Comparison McCoy:Truview EVO2
Evidence: indirect

NMA estimate: **-8.027**
95% intervals for NMA estimate
Confidence interval: **(-22.235,6.182)**
Prediction interval: **(-30.660,14.606)**
Prediction interval extends into clinically important or unimportant effects

Heterogeneity judgment
Some concerns

Comparison McCoy:Wis-Hipple
Evidence: indirect

NMA estimate: **1.306**
95% intervals for NMA estimate
Confidence interval:
Prediction interval: **(-16.913,19.525)**
Confidence and **(-25.432,28.044)**
prediction intervals agree in relation to clinically important effect

Heterogeneity judgment
No concerns

Comparison McGrath:Miller
Evidence: indirect

NMA estimate: **9.125**
95% intervals for NMA estimate
Confidence interval: **(-9.913,28.164)**
Prediction interval: **(-18.488,36.739)**
Prediction interval extends into clinically important or unimportant effects

Heterogeneity judgment
Some concerns

Comparison McGrath:Truview EVO2
Evidence: indirect

NMA estimate: **-1.121**
95% intervals for NMA estimate
Confidence interval:
Prediction interval: **(-20.387,18.144)**
Confidence and **(-28.980,26.737)**
prediction intervals agree in relation to clinically important effect

Heterogeneity judgment
No concerns

Comparison McGrath:Wis-Hipple
Evidence: indirect

NMA estimate: **8.211**
95% intervals for NMA estimate
Confidence interval:
Prediction interval: **(-15.761,32.184)**
Confidence and **(-24.854,41.277)**
prediction intervals agree in relation to clinically important effect

Heterogeneity judgment
No concerns

Comparison Miller:Truview EVO2
Evidence: indirect

NMA estimate: **-10.247**
95% intervals for NMA estimate
Confidence interval: **(-23.661,3.167)**
Prediction interval: **(-32.111,11.617)**
Prediction interval extends into clinically important or unimportant effects

Heterogeneity judgment
Some concerns

Comparison
Evidence: Truview EVO2:Wis-Hipple
indirect

NMA estimate: **9.333**
95% intervals for NMA estimate
Confidence interval:
Prediction interval: **(-10.471,29.137)**
Confidence and **(-19.107,37.773)**
prediction intervals agree in relation to clinically important effect

Heterogeneity judgment
No concerns

Supplemental S9 Results of funnel plot of the heart rate (A) mean blood pressure (B), and intubation time (C).

A. Heart rate

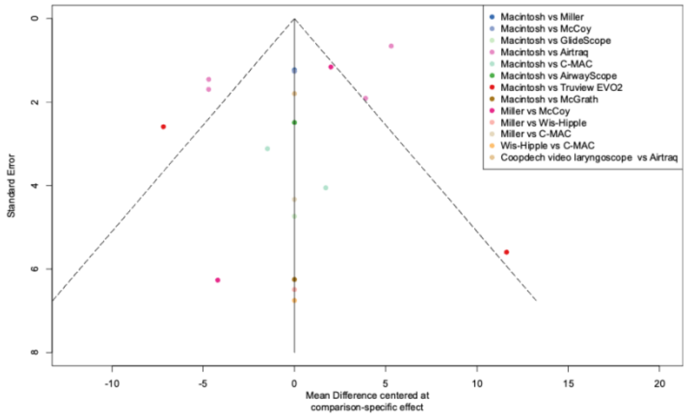

B. Mean blood pressure

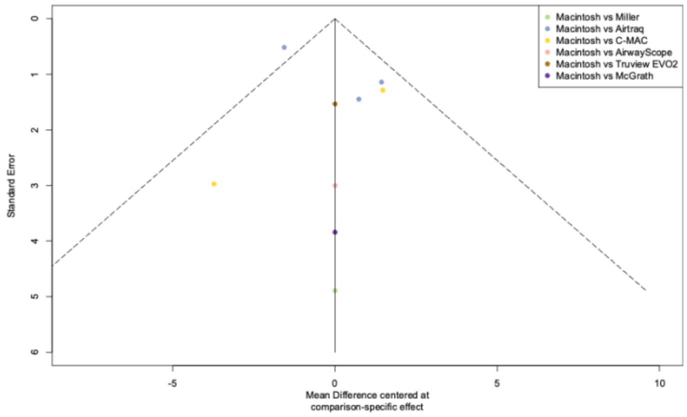

C. Intubation time

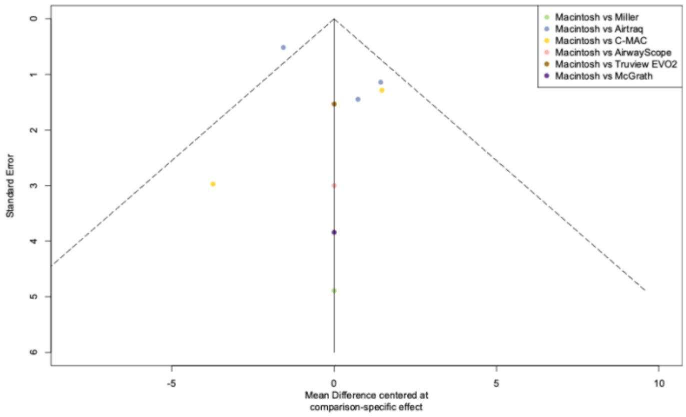

Supplemental S10 League table of the mean blood pressure.

**Airtraq**	.	.	-8.6 (-10.9; -6.3)	.	.	.
-13.6 (-20.7; -6.4)	**AirwayScope**	.	5.0 (-1.8; 11.8)	.	.	.
-12.0 (-16.3; -7.8)	1.5 (-6.2; 9.2)	**C-MAC**	3.5 (-0.1; 7.1)	.	.	.
-8.6 (-10.9; -6.3)	5.0 (-1.8; 11.8)	3.5 (-0.1; 7.1)	**Macintosh**	-6.0 (-14.3; 2.3)	-9.5 (-19.7; 0.7)	-0.3 (-4.8; 4.2)
-14.6 (-23.1; -6.0)	-1.0 (-11.7; 9.7)	-2.5 (-11.5; 6.5)	-6.0 (-14.3; 2.3)	**McGrath**	.	.
-18.1 (-28.5; -7.6)	-4.5 (-16.7; 7.7)	-6.0 (-16.8; 4.8)	-9.5 (-19.7; 0.7)	-3.5 (-16.6; 9.6)	**Miller**	.
-8.9 (-14.0; -3.8)	4.7 (-3.5; 12.9)	3.2 (-2.6; 9.0)	-0.3 (-4.8; 4.2)	5.7 (-3.7; 15.1)	9.2 (-1.9; 20.3)	**Truview EVO2**

Supplemental S11 Results of imprecision of the mean blood pressure.

Comparison **Airtraq:Macintosh**
Evidence: mixed
NMA estimate: **-8.566**
95% Confidence interval:
Confidence interval **(-10.862,-6.269)** *does not cross clinically important effect*
Imprecision judgment
No concerns

Comparison
Evidence: **AirwayScope:Macintosh**
mixed
NMA estimate: **5.000**
95% Confidence interval:
Confidence interval **(-1.804,11.804)** *extends into clinically important effects*
Imprecision judgment
Some concerns

Comparison **C-MAC:Macintosh**
Evidence: mixed
NMA estimate: **3.472**
95% Confidence interval:
Confidence interval **(-0.121,7.065)** *extends into clinically important effects*
Imprecision judgment
Some concerns

Comparison **Macintosh:McGrath**
Evidence: mixed
NMA estimate: **-6.000**
95% Confidence interval:
Confidence interval **(-14.262,2.262)** *extends into clinically important effects*
Imprecision judgment
Some concerns

Comparison **Macintosh:Miller**
Evidence: mixed
NMA estimate: **-9.500**
95% Confidence interval:
Confidence interval **(-19.674,0.674)** *extends into clinically important effects*
Imprecision judgment
Some concerns

Comparison
Evidence: **Macintosh:Truview EVO2**
mixed
NMA estimate: **-0.300**
95% Confidence interval:
Confidence interval **(-4.850,4.250)** *does not cross clinically important effect*
Imprecision judgment
No concerns

Comparison **Airtraq:AirwayScope**
Evidence: indirect
NMA estimate: **-13.566**
95% Confidence interval:
Confidence interval **(-20.747,-6.384)** *does not cross clinically important effect*
Imprecision judgment
No concerns

Comparison **Airtraq:C-MAC**
Evidence: indirect
NMA estimate: **-12.038**
95% Confidence interval:
Confidence interval **(-16.302,-7.774)** *does not cross clinically important effect*
Imprecision judgment
No concerns

Comparison **Airtraq:McGrath**
Evidence: indirect
NMA estimate: **-14.566**
95% Confidence interval:
Confidence interval **(-23.141,-5.990)** *does not cross clinically important effect*
Imprecision judgment
No concerns

Comparison **Airtraq:Miller**
Evidence: indirect
NMA estimate: **-18.066**
95% Confidence interval:
Confidence interval **(-28.496,-7.635)** *does not cross clinically important effect*
Imprecision judgment
No concerns

Comparison **Airtraq:Truview EVO2**
Evidence: indirect
NMA estimate: **-8.866**
95% Confidence interval:
Confidence interval **(-13.962,-3.769)** *does not cross clinically important effect*
Imprecision judgment
No concerns

Comparison **AirwayScope:C-MAC**
Evidence: indirect
NMA estimate: **1.528**
95% Confidence interval:
Confidence interval **(-6.166,9.222)** *extends into clinically important effects in both directions*
Imprecision judgment
Major concerns

Comparison **AirwayScope:McGrath**
Evidence: indirect
NMA estimate: **-1.000**
95% Confidence interval:
Confidence interval **(-11.703,9.703)** *extends into clinically important effects in both directions*
Imprecision judgment
Major concerns

Comparison **AirwayScope:Miller**
Evidence: indirect
NMA estimate: **-4.500**
95% Confidence interval:
Confidence interval **(-16.740,7.740)** *extends into clinically important effects in both directions*
Imprecision judgment
Major concerns

Comparison **AirwayScope:Truview EVO2**
Evidence: indirect
NMA estimate: **4.700**
95% Confidence interval:
Confidence interval **(-3.485,12.885)** *extends into clinically important effects*
Imprecision judgment
Some concerns

Comparison **C-MAC:McGrath**
Evidence: indirect
NMA estimate: **-2.528**
95% Confidence interval:
Confidence interval **(-11.537,6.481)** *extends into clinically important effects in both directions*
Imprecision judgment
Major concerns

Comparison **C-MAC:Miller**
Evidence: indirect
NMA estimate: **-6.028**
95% Confidence interval:
Confidence interval **(-16.818,4.762)** *extends into clinically important effects*
Imprecision judgment
Some concerns

Comparison **C-MAC:Truview EVO2**
Evidence: indirect
NMA estimate: **3.172**
95% Confidence interval:
Confidence interval **(-2.625,8.970)** *extends into clinically important effects*
Imprecision judgment
Some concerns

Comparison **McGrath:Miller**
Evidence: indirect
NMA estimate: **-3.500**
95% Confidence interval:
Confidence interval **(-16.607,9.607)** *extends into clinically important effects in both directions*
Imprecision judgment
Major concerns

Comparison **McGrath:Truview EVO2**
Evidence: indirect
NMA estimate: **5.700**
95% Confidence interval:
Confidence interval **(-3.732,15.132)** *extends into clinically important effects*
Imprecision judgment
Some concerns

Comparison **Miller:Truview EVO2**
Evidence: indirect
NMA estimate: **9.200**
95% Confidence interval:
Confidence interval **(-1.945,20.345)** *extends into clinically important effects*
Imprecision judgment
Some concerns

Supplemental S12 Results of heterogeneity of the mean blood pressure.

Comparison **Airtraq:Macintosh**
Evidence: mixed

NMA estimate: **-8.566**
95% intervals for NMA estimate
Confidence interval: **(-10.862,-6.269)**
Prediction interval: **(-17.600,0.469)**

Prediction interval extends into clinically important or unimportant effects

Heterogeneity judgment
Some concerns

Comparison
Evidence: AirwayScope:Macintosh
mixed

NMA estimate: **5.000**
95% intervals for NMA estimate
Confidence interval: **(-1.804,11.804)**
Prediction interval: **(-11.712,21.712)**

Prediction interval extends into clinically important or unimportant effects

Heterogeneity judgment
Some concerns

Comparison **C-MAC:Macintosh**
Evidence: mixed

NMA estimate: **3.472**
95% intervals for NMA estimate
Confidence interval: **(-0.121,7.065)**
Prediction interval: **(-7.410,14.354)**

Prediction interval extends into clinically important or unimportant effects

Heterogeneity judgment
Some concerns

Comparison **Macintosh:McGrath**
Evidence: mixed

NMA estimate: **-6.000**
95% intervals for NMA estimate
Confidence interval: **(-14.262,2.262)**
Prediction interval: **(-25.626,13.626)**

Prediction interval extends into clinically important or unimportant effects

Heterogeneity judgment
Some concerns

Comparison **Macintosh:Miller**
Evidence: mixed

NMA estimate: **-9.500**
95% intervals for NMA estimate
Confidence interval: **(-19.674,0.674)**
Prediction interval: **(-33.060,14.060)**

Prediction interval extends into clinically important or unimportant effects

Heterogeneity judgment
Some concerns

Comparison
Evidence: Macintosh:Truview EVO2
mixed

NMA estimate: **-0.300**
95% intervals for NMA estimate
Confidence interval: **(-4.850,4.250)**
Prediction interval: **(-12.789,12.189)**

Prediction interval extends into clinically important effects in both directions

Heterogeneity judgment
Major concerns

Comparison **Airtraq:AirwayScope**
Evidence: indirect

NMA estimate: **-13.566**
95% intervals for NMA estimate
Confidence interval: **(-20.747,-6.384)**
Prediction interval: **(-31.022,3.891)**

Prediction interval extends into clinically important or unimportant effects

Heterogeneity judgment
Some concerns

Comparison **Airtraq:C-MAC**
Evidence: indirect

NMA estimate: **-12.038**
95% intervals for NMA estimate
Confidence interval: **(-16.302,-7.774)**
Prediction interval: **(-24.031,-0.045)**

Confidence and prediction intervals agree in relation to clinically important effect

Heterogeneity judgment
No concerns

Comparison **Airtraq:McGrath**
Evidence: indirect

NMA estimate: **-14.566**
95% intervals for NMA estimate
Confidence interval: **(-23.141,-5.990)**
Prediction interval: **(-34.828,5.697)**

Prediction interval extends into clinically important effects in both directions

Heterogeneity judgment
Major concerns

Comparison **Airtraq:Miller**
Evidence: indirect

NMA estimate: **-18.066**
95% intervals for NMA estimate
Confidence interval: **(-28.496,-7.635)**
Prediction interval: **(-42.159,6.028)**

Prediction interval extends into clinically important effects in both directions

Heterogeneity judgment
Major concerns

Comparison **Airtraq:Truview EVO2**
Evidence: indirect

NMA estimate: **-8.866**
95% intervals for NMA estimate
Confidence interval: **(-13.962,-3.769)**
Prediction interval: **(-22.333,4.603)**

Prediction interval extends into clinically important or unimportant effects

Heterogeneity judgment
Some concerns

Comparison **AirwayScope:C-MAC**
Evidence: indirect

NMA estimate: **1.528**
95% intervals for NMA estimate
Confidence interval: **(-6.166,9.222)**
Prediction interval: **(-16.952,20.008)**

Confidence and prediction intervals agree in relation to clinically important effect

Heterogeneity judgment
No concerns

Comparison AirwayScope:McGrath
Evidence: indirect
NMA estimate: **-1.000**
95% intervals for NMA estimate
Confidence interval: **(-11.703,9.703)**
Prediction interval: **(-25.663,23.663)**
Confidence and prediction intervals agree in relation to clinically important effect
Heterogeneity judgment
No concerns

Comparison AirwayScope:Miller
Evidence: indirect
NMA estimate: **-4.500**
95% intervals for NMA estimate
Confidence interval: **(-16.740,7.740)**
Prediction interval: **(-32.396,23.396)**
Confidence and prediction intervals agree in relation to clinically important effect
Heterogeneity judgment
No concerns

Comparison AirwayScope:Truview EVO2
Evidence: indirect
NMA estimate: **4.700**
95% intervals for NMA estimate
Confidence interval: **(-3.485,12.885)**
Prediction interval: **(-14.770,24.169)**
Prediction interval extends into clinically important or unimportant effects
Heterogeneity judgment
Some concerns

Comparison C-MAC:McGrath
Evidence: indirect
NMA estimate: **-2.528**
95% intervals for NMA estimate
Confidence interval: **(-11.537,6.481)**
Prediction interval: **(-23.679,18.623)**
Confidence and prediction intervals agree in relation to clinically important effect
Heterogeneity judgment
No concerns

Comparison C-MAC:Miller
Evidence: indirect
NMA estimate: **-6.028**
95% intervals for NMA estimate
Confidence interval: **(-16.818,4.762)**
Prediction interval: **(-30.873,18.818)**
Prediction interval extends into clinically important or unimportant effects
Heterogeneity judgment
Some concerns

Comparison C-MAC:Truview EVO2
Evidence: indirect
NMA estimate: **3.172**
95% intervals for NMA estimate
Confidence interval: **(-2.625,8.970)**
Prediction interval: **(-11.599,17.943)**
Prediction interval extends into clinically important or unimportant effects
Heterogeneity judgment
Some concerns

Comparison McGrath:Miller
Evidence: indirect
NMA estimate: **-3.500**
95% intervals for NMA estimate
Confidence interval: **(-16.607,9.607)**
Prediction interval: **(-33.233,26.233)**
Confidence and prediction intervals agree in relation to clinically important effect
Heterogeneity judgment
No concerns

Comparison McGrath:Truview EVO2
Evidence: indirect
NMA estimate: **5.700**
95% intervals for NMA estimate
Confidence interval: **(-3.732,15.132)**
Prediction interval: **(-16.321,27.721)**
Prediction interval extends into clinically important or unimportant effects
Heterogeneity judgment
Some concerns

Comparison Miller:Truview EVO2
Evidence: indirect
NMA estimate: **9.200**
95% intervals for NMA estimate
Confidence interval: **(-1.945,20.345)**
Prediction interval: **(-16.390,34.790)**
Prediction interval extends into clinically important or unimportant effects
Heterogeneity judgment
Some concerns

Supplemental S13 League table of the intubation time.

**Airtraq**	.	.	-5.1 (-14.1; 3.9)	.	-12.6 (-18.4; -6.8)	.	.	-3.6 (-12.2; 5.0)	.	.
-11.7 (-21.8; -1.6)	**AirwayScope**	.	.	.	0.5 (-8.2; 9.2)	.	.	.	.	.
-6.3 (-17.3; 4.8)	5.5 (-8.6; 19.5)	**C-MAC**	.	.	.	.	.	-0.4 (-9.2; 8.4)	.	-1.6 (-10.4; 7.2)
-5.1 (-14.1; 3.9)	6.6 (-6.9; 20.1)	1.2 (-13.1; 15.4)	**Coopdech video laryngoscope**	.	.	.	.	.	.	.
-16.7 (-27.2; -6.2)	-5.0 (-17.6; 7.6)	-10.5 (-24.8; 3.9)	-11.6 (-25.4; 2.2)	**GlideScope**	5.5 (-3.6; 14.6)	.	.	.	.	.
-11.2 (-16.4; -6.0)	0.5 (-8.2; 9.2)	-5.0 (-16.1; 6.1)	-6.1 (-16.5; 4.2)	5.5 (-3.6; 14.6)	**Macintosh**	-0.5 (-9.3; 8.3)	0.5 (-6.2; 7.2)	1.2 (-7.8; 10.2)	.	.
-10.1 (-19.2; -1.0)	1.6 (-10.4; 13.7)	-3.8 (-16.0; 8.3)	-5.0 (-17.8; 7.8)	6.6 (-5.7; 19.0)	1.1 (-7.2; 9.5)	**McCoy**	.	1.7 (-7.3; 10.7)	.	.
-10.7 (-19.2; -2.2)	1.0 (-9.9; 11.9)	-4.5 (-17.4; 8.5)	-5.6 (-18.0; 6.7)	6.0 (-5.3; 17.3)	0.5 (-6.2; 7.2)	-0.6 (-11.3; 10.1)	**McGrath**	.	.	.
-6.7 (-13.3; 0.0)	5.1 (-5.9; 16.1)	-0.4 (-9.2; 8.4)	-1.6 (-12.7; 9.6)	10.1 (-1.3; 21.5)	4.6 (-2.2; 11.4)	3.4 (-5.0; 11.9)	4.1 (-5.5; 13.6)	**Miller**	-7.4 (-16.6; 1.7)	-1.2 (-10.0; 7.6)
-14.1 (-25.4; -2.8)	-2.4 (-16.7; 11.9)	-7.8 (-20.5; 4.8)	-9.0 (-23.4; 5.4)	2.6 (-12.0; 17.2)	-2.9 (-14.2; 8.5)	-4.0 (-16.4; 8.4)	-3.4 (-16.6; 9.8)	-7.4 (-16.6; 1.7)	**Truview EVO2**	.
-7.9 (-18.9; 3.2)	3.9 (-10.2; 18.0)	-1.6 (-10.4; 7.2)	-2.8 (-17.0; 11.5)	8.9 (-5.5; 23.3)	3.4 (-7.8; 14.5)	2.2 (-10.0; 14.5)	2.9 (-10.1; 15.9)	-1.2 (-10.0; 7.6)	6.2 (-6.5; 18.9)	**Wis-Hipple**

Supplemental S14 Results of inconsistency of the intubation time.

Comparison	P value
McCoy vs Macintosh	0.27
Airtraq vs Macintosh	0.27
Miller vs Macintosh	0.27
Airtraq vs Miller	0.27
McCoy vs Miller	0.27

Supplemental S15 Results of imprecision of the intubation time.

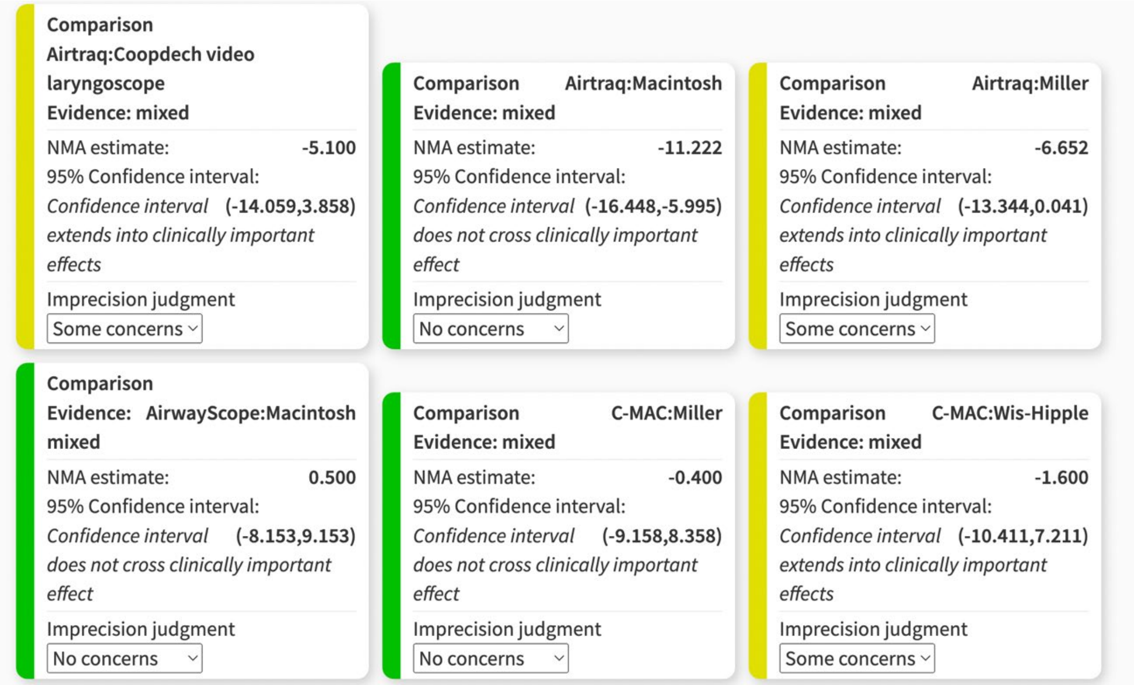

Comparison GlideScope:Macintosh
Evidence: mixed
NMA estimate: **5.500**
95% Confidence interval: **(-3.630,14.630)**
Confidence interval extends into clinically important effects
Imprecision judgment: Some concerns

Comparison Macintosh:McCoy
Evidence: mixed
NMA estimate: **1.128**
95% Confidence interval: **(-7.223,9.478)**
Confidence interval does not cross clinically important effect
Imprecision judgment: No concerns

Comparison Macintosh:McGrath
Evidence: mixed
NMA estimate: **0.503**
95% Confidence interval: **(-6.198,7.204)**
Confidence interval does not cross clinically important effect
Imprecision judgment: No concerns

Comparison Macintosh:Miller
Evidence: mixed
NMA estimate: **4.570**
95% Confidence interval: **(-2.223,11.363)**
Confidence interval extends into clinically important effects
Imprecision judgment: Some concerns

Comparison McCoy:Miller
Evidence: mixed
NMA estimate: **3.442**
95% Confidence interval: **(-5.001,11.886)**
Confidence interval extends into clinically important effects
Imprecision judgment: Some concerns

Comparison Miller:Truview EVO2
Evidence: mixed
NMA estimate: **-7.440**
95% Confidence interval: **(-16.569,1.689)**
Confidence interval extends into clinically important effects
Imprecision judgment: Some concerns

Comparison Miller:Wis-Hipple
Evidence: mixed
NMA estimate: **-1.200**
95% Confidence interval: **(-10.026,7.626)**
Confidence interval extends into clinically important effects
Imprecision judgment: Some concerns

Comparison Airtraq:AirwayScope
Evidence: indirect
NMA estimate: **-11.722**
95% Confidence interval: **(-21.830,-1.613)**
Confidence interval does not cross clinically important effect
Imprecision judgment: No concerns

Comparison Airtraq:C-MAC
Evidence: indirect
NMA estimate: **-6.252**
95% Confidence interval: **(-17.274,4.771)**
Confidence interval extends into clinically important effects
Imprecision judgment: Some concerns

Comparison Airtraq:GlideScope
Evidence: indirect
NMA estimate: **-16.722**
95% Confidence interval: **(-27.242,-6.202)**
Confidence interval does not cross clinically important effect
Imprecision judgment: No concerns

Comparison Airtraq:McCoy
Evidence: indirect
NMA estimate: **-10.094**
95% Confidence interval: **(-19.215,-0.973)**
Confidence interval does not cross clinically important effect
Imprecision judgment: No concerns

Comparison Airtraq:McGrath
Evidence: indirect
NMA estimate: **-10.719**
95% Confidence interval: **(-19.217,-2.220)**
Confidence interval does not cross clinically important effect
Imprecision judgment: No concerns

Comparison **Airtraq:Truview EVO2**
Evidence: indirect

NMA estimate: **-14.092**
95% Confidence interval:
Confidence interval **(-25.411,-2.772)**
does not cross clinically important effect

Imprecision judgment
No concerns

Comparison **Airtraq:Wis-Hipple**
Evidence: indirect

NMA estimate: **-7.852**
95% Confidence interval:
Confidence interval **(-18.928,3.225)**
extends into clinically important effects

Imprecision judgment
Some concerns

Comparison **AirwayScope:C-MAC**
Evidence: indirect

NMA estimate: **5.470**
95% Confidence interval:
Confidence interval **(-8.591,19.531)**
extends into clinically important effects

Imprecision judgment
Some concerns

Comparison
AirwayScope:Coopdech video laryngoscope
Evidence: indirect

NMA estimate: **6.622**
95% Confidence interval:
Confidence interval **(-6.885,20.129)**
extends into clinically important effects

Imprecision judgment
Some concerns

Comparison
Evidence: AirwayScope:GlideScope indirect

NMA estimate: **-5.000**
95% Confidence interval:
Confidence interval **(-17.579,7.579)**
extends into clinically important effects

Imprecision judgment
Some concerns

Comparison **AirwayScope:McCoy**
Evidence: indirect

NMA estimate: **1.628**
95% Confidence interval:
Confidence interval **(-10.397,13.652)**
extends into clinically important effects in both directions

Imprecision judgment
Major concerns

Comparison **AirwayScope:McGrath**
Evidence: indirect

NMA estimate: **1.003**
95% Confidence interval:
Confidence interval **(-9.941,11.947)**
extends into clinically important effects

Imprecision judgment
Some concerns

Comparison **AirwayScope:Miller**
Evidence: indirect

NMA estimate: **5.070**
95% Confidence interval:
Confidence interval **(-5.931,16.071)**
extends into clinically important effects

Imprecision judgment
Some concerns

Comparison
AirwayScope:Truview EVO2
Evidence: indirect

NMA estimate: **-2.370**
95% Confidence interval:
Confidence interval **(-16.665,11.925)**
extends into clinically important effects in both directions

Imprecision judgment
Major concerns

Comparison
Evidence: AirwayScope:Wis-Hipple indirect

NMA estimate: **3.870**
95% Confidence interval:
Confidence interval **(-10.234,17.974)**
extends into clinically important effects in both directions

Imprecision judgment
Major concerns

Comparison
C-MAC:Coopdech video laryngoscope
Evidence: indirect

NMA estimate: **1.152**
95% Confidence interval:
Confidence interval **(-13.052,15.355)**
extends into clinically important effects in both directions

Imprecision judgment
Major concerns

Comparison **C-MAC:GlideScope**
Evidence: indirect

NMA estimate: **-10.470**
95% Confidence interval:
Confidence interval **(-24.830,3.890)**
extends into clinically important effects

Imprecision judgment
Some concerns

Comparison C-MAC:Macintosh
Evidence: indirect

NMA estimate: **-4.970**
95% Confidence interval:
Confidence interval **(-16.054,6.114)** *extends into clinically important effects*

Imprecision judgment
Some concerns

Comparison C-MAC:McCoy
Evidence: indirect

NMA estimate: **-3.842**
95% Confidence interval:
Confidence interval **(-16.008,8.323)** *extends into clinically important effects*

Imprecision judgment
Some concerns

Comparison C-MAC:McGrath
Evidence: indirect

NMA estimate: **-4.467**
95% Confidence interval:
Confidence interval **(-17.419,8.485)** *extends into clinically important effects*

Imprecision judgment
Some concerns

Comparison C-MAC:Truview EVO2
Evidence: indirect

NMA estimate: **-7.840**
95% Confidence interval:
Confidence interval **(-20.491,4.811)** *extends into clinically important effects*

Imprecision judgment
Some concerns

Comparison Coopdech video laryngoscope:GlideScope
Evidence: indirect

NMA estimate: **-11.622**
95% Confidence interval:
Confidence interval **(-25.439,2.196)** *extends into clinically important effects*

Imprecision judgment
Some concerns

Comparison Coopdech video laryngoscope:Macintosh
Evidence: indirect

NMA estimate: **-6.122**
95% Confidence interval:
Confidence interval **(-16.493,4.250)** *extends into clinically important effects*

Imprecision judgment
Some concerns

Comparison Coopdech video laryngoscope:McCoy
Evidence: indirect

NMA estimate: **-4.994**
95% Confidence interval:
Confidence interval **(-17.779,7.791)** *extends into clinically important effects*

Imprecision judgment
Some concerns

Comparison Coopdech video laryngoscope:McGrath
Evidence: indirect

NMA estimate: **-5.619**
95% Confidence interval:
Confidence interval **(-17.967,6.730)** *extends into clinically important effects*

Imprecision judgment
Some concerns

Comparison Coopdech video laryngoscope:Miller
Evidence: indirect

NMA estimate: **-1.552**
95% Confidence interval:
Confidence interval **(-12.734,9.631)** *extends into clinically important effects*

Imprecision judgment
Some concerns

Comparison Coopdech video laryngoscope:Truview EVO2
Evidence: indirect

NMA estimate: **-8.992**
95% Confidence interval:
Confidence interval **(-23.427,5.444)** *extends into clinically important effects*

Imprecision judgment
Some concerns

Comparison Coopdech video laryngoscope:Wis-Hipple
Evidence: indirect

NMA estimate: **-2.752**
95% Confidence interval:
Confidence interval **(-16.997,11.494)** *extends into clinically important effects in both directions*

Imprecision judgment
Major concerns

Comparison GlideScope:McCoy
Evidence: indirect

NMA estimate: **6.628**
95% Confidence interval:
Confidence interval **(-5.745,19.000)** *extends into clinically important effects*

Imprecision judgment
Some concerns

Comparison GlideScope:McGrath
Evidence: indirect

NMA estimate: **6.003**
95% Confidence interval:
Confidence interval **(-5.322,17.328)** *extends into clinically important effects*

Imprecision judgment
Some concerns

Comparison GlideScope:Miller
Evidence: indirect

NMA estimate: **10.070**
95% Confidence interval:
Confidence interval **(-1.310,21.450)** *extends into clinically important effects*

Imprecision judgment
Some concerns

Comparison GlideScope:Truview EVO2
Evidence: indirect

NMA estimate: **2.630**
95% Confidence interval:
Confidence interval **(-11.959,17.219)** *extends into clinically important effects in both directions*

Imprecision judgment
Major concerns

Comparison GlideScope:Wis-Hipple
Evidence: indirect

NMA estimate: **8.870**
95% Confidence interval:
Confidence interval **(-5.531,23.272)** *extends into clinically important effects*

Imprecision judgment
Some concerns

Comparison Macintosh:Truview EVO2
Evidence: indirect

NMA estimate: **-2.870**
95% Confidence interval:
Confidence interval **(-14.249,8.509)** *extends into clinically important effects*

Imprecision judgment
Some concerns

Comparison Macintosh:Wis-Hipple
Evidence: indirect

NMA estimate: **3.370**
95% Confidence interval:
Confidence interval **(-7.768,14.508)** *extends into clinically important effects*

Imprecision judgment
Some concerns

Comparison McCoy:McGrath
Evidence: indirect

NMA estimate: **-0.625**
95% Confidence interval:
Confidence interval **(-11.331,10.082)** *extends into clinically important effects in both directions*

Imprecision judgment
Major concerns

Comparison McCoy:Truview EVO2
Evidence: indirect

NMA estimate: **-3.998**
95% Confidence interval:
Confidence interval **(-16.433,8.438)** *extends into clinically important effects*

Imprecision judgment
Some concerns

Comparison McCoy:Wis-Hipple
Evidence: indirect

NMA estimate: **2.242**
95% Confidence interval:
Confidence interval **(-9.972,14.457)** *extends into clinically important effects*

Imprecision judgment
Some concerns

Comparison McGrath:Miller
Evidence: indirect

NMA estimate: **4.067**
95% Confidence interval:
Confidence interval **(-5.475,13.609)** *extends into clinically important effects*

Imprecision judgment
Some concerns

Comparison McGrath:Truview EVO2
Evidence: indirect

NMA estimate: **-3.373**
95% Confidence interval:
Confidence interval **(-16.579,9.832)** *extends into clinically important effects*

Imprecision judgment
Some concerns

Comparison McGrath:Wis-Hipple
Evidence: indirect

NMA estimate: **2.867**
95% Confidence interval:
Confidence interval **(-10.131,15.865)** *extends into clinically important effects in both directions*

Imprecision judgment
Major concerns

Comparison Truview EVO2:Wis-Hipple
Evidence: indirect

NMA estimate: **6.240**
95% Confidence interval:
Confidence interval **(-6.458,18.938)** *extends into clinically important effects*

Imprecision judgment
Some concerns

Supplemental S16 Results of heterogeneity of the intubation time.

Comparison
Airtraq:Coopdech video laryngoscope
Evidence: mixed
NMA estimate: **-5.100**
95% intervals for NMA estimate
Confidence interval: **(-14.059,3.858)**
Prediction interval: **(-25.224,15.024)**
Prediction interval extends into clinically important or unimportant effects
Heterogeneity judgment
Some concerns

Comparison Airtraq:Macintosh
Evidence: mixed
NMA estimate: **-11.222**
95% intervals for NMA estimate
Confidence interval:**(-16.448,-5.995)**
Prediction interval: **(-27.513,5.069)**
Prediction interval extends into clinically important or unimportant effects
Heterogeneity judgment
Some concerns

Comparison Airtraq:Miller
Evidence: mixed
NMA estimate: **-6.652**
95% intervals for NMA estimate
Confidence interval: **(-13.344,0.041)**
Prediction interval: **(-24.300,10.997)**
Prediction interval extends into clinically important or unimportant effects
Heterogeneity judgment
Some concerns

Comparison
Evidence: AirwayScope:Macintosh mixed
NMA estimate: **0.500**
95% intervals for NMA estimate
Confidence interval: **(-8.153,9.153)**
Prediction interval: **(-19.268,20.268)**
Prediction interval extends into clinically important effects in both directions
Heterogeneity judgment
Major concerns

Comparison C-MAC:Miller
Evidence: mixed
NMA estimate: **-0.400**
95% intervals for NMA estimate
Confidence interval: **(-9.158,8.358)**
Prediction interval: **(-20.290,19.490)**
Prediction interval extends into clinically important effects in both directions
Heterogeneity judgment
Major concerns

Comparison C-MAC:Wis-Hipple
Evidence: mixed
NMA estimate: **-1.600**
95% intervals for NMA estimate
Confidence interval: **(-10.411,7.211)**
Prediction interval: **(-21.551,18.351)**
Prediction interval extends into clinically important or unimportant effects
Heterogeneity judgment
Some concerns

Comparison GlideScope:Macintosh
Evidence: mixed
NMA estimate: **5.500**
95% intervals for NMA estimate
Confidence interval: **(-3.630,14.630)**
Prediction interval: **(-14.826,25.826)**
Prediction interval extends into clinically important or unimportant effects
Heterogeneity judgment
Some concerns

Comparison Macintosh:McCoy
Evidence: mixed
NMA estimate: **1.128**
95% intervals for NMA estimate
Confidence interval: **(-7.223,9.478)**
Prediction interval: **(-18.294,20.550)**
Prediction interval extends into clinically important effects in both directions
Heterogeneity judgment
Major concerns

Comparison Macintosh:McGrath
Evidence: mixed
NMA estimate: **0.503**
95% intervals for NMA estimate
Confidence interval: **(-6.198,7.204)**
Prediction interval: **(-17.154,18.160)**
Prediction interval extends into clinically important effects in both directions
Heterogeneity judgment
Major concerns

Comparison Macintosh:Miller
Evidence: mixed
NMA estimate: **4.570**
95% intervals for NMA estimate
Confidence interval: **(-2.223,11.363)**
Prediction interval: **(-13.180,22.320)**
Prediction interval extends into clinically important or unimportant effects
Heterogeneity judgment
Some concerns

Comparison McCoy:Miller
Evidence: mixed
NMA estimate: **3.442**
95% intervals for NMA estimate
Confidence interval: **(-5.001,11.886)**
Prediction interval: **(-16.086,22.971)**
Prediction interval extends into clinically important or unimportant effects
Heterogeneity judgment
Some concerns

Comparison Miller:Truview EVO2
Evidence: mixed
NMA estimate: **-7.440**
95% intervals for NMA estimate
Confidence interval: **(-16.569,1.689)**
Prediction interval: **(-27.765,12.885)**
Prediction interval extends into clinically important or unimportant effects
Heterogeneity judgment
Some concerns

Comparison **Miller:Wis-Hipple**
Evidence: mixed

NMA estimate: **-1.200**
95% intervals for NMA estimate
Confidence interval: **(-10.026,7.626)**
Prediction interval: **(-21.169,18.769)**

Prediction interval extends into clinically important or unimportant effects

Heterogeneity judgment
Some concerns

Comparison **Airtraq:AirwayScope**
Evidence: indirect

NMA estimate: **-11.722**
95% intervals for NMA estimate
Confidence interval:**(-21.830,-1.613)**
Prediction interval: **(-33.234,9.791)**

Prediction interval extends into clinically important or unimportant effects

Heterogeneity judgment
Some concerns

Comparison **Airtraq:C-MAC**
Evidence: indirect

NMA estimate: **-6.252**
95% intervals for NMA estimate
Confidence interval: **(-17.274,4.771)**
Prediction interval: **(-28.917,16.413)**

Prediction interval extends into clinically important or unimportant effects

Heterogeneity judgment
Some concerns

Comparison **Airtraq:GlideScope**
Evidence: indirect

NMA estimate: **-16.722**
95% intervals for NMA estimate
Confidence interval:**(-27.242,-6.202)**
Prediction interval: **(-38.748,5.305)**

Prediction interval extends into clinically important or unimportant effects

Heterogeneity judgment
Some concerns

Comparison **Airtraq:McCoy**
Evidence: indirect

NMA estimate: **-10.094**
95% intervals for NMA estimate
Confidence interval:**(-19.215,-0.973)**
Prediction interval: **(-30.410,10.222)**

Prediction interval extends into clinically important effects in both directions

Heterogeneity judgment
Major concerns

Comparison **Airtraq:McGrath**
Evidence: indirect

NMA estimate: **-10.719**
95% intervals for NMA estimate
Confidence interval:**(-19.217,-2.220)**
Prediction interval: **(-30.309,8.872)**

Prediction interval extends into clinically important or unimportant effects

Heterogeneity judgment
Some concerns

Comparison **Airtraq:Truview EVO2**
Evidence: indirect

NMA estimate: **-14.092**
95% intervals for NMA estimate
Confidence interval:**(-25.411,-2.772)**
Prediction interval: **(-37.139,8.956)**

Prediction interval extends into clinically important or unimportant effects

Heterogeneity judgment
Some concerns

Comparison **Airtraq:Wis-Hipple**
Evidence: indirect

NMA estimate: **-7.852**
95% intervals for NMA estimate
Confidence interval: **(-18.928,3.225)**
Prediction interval: **(-30.586,14.883)**

Prediction interval extends into clinically important or unimportant effects

Heterogeneity judgment
Some concerns

Comparison **AirwayScope:C-MAC**
Evidence: indirect

NMA estimate: **5.470**
95% intervals for NMA estimate
Confidence interval: **(-8.591,19.531)**
Prediction interval: **(-21.263,32.203)**

Prediction interval extends into clinically important or unimportant effects

Heterogeneity judgment
Some concerns

Comparison
AirwayScope:Coopdech video laryngoscope
Evidence: indirect

NMA estimate: **6.622**
95% intervals for NMA estimate
Confidence interval: **(-6.885,20.129)**
Prediction interval: **(-19.347,32.591)**

Prediction interval extends into clinically important or unimportant effects

Heterogeneity judgment
Some concerns

Comparison
Evidence: AirwayScope:GlideScope
indirect

NMA estimate: **-5.000**
95% intervals for NMA estimate
Confidence interval: **(-17.579,7.579)**
Prediction interval: **(-29.709,19.709)**

Prediction interval extends into clinically important or unimportant effects

Heterogeneity judgment
Some concerns

Comparison **AirwayScope:McCoy**
Evidence: indirect

NMA estimate: **1.628**
95% intervals for NMA estimate
Confidence interval:
Prediction interval: **(-10.397,13.652)**
Confidence and **(-22.343,25.599)**
prediction intervals agree in relation to clinically important effect

Heterogeneity judgment
No concerns

Comparison AirwayScope:McGrath
Evidence: indirect

NMA estimate: **1.003**

95% intervals for NMA estimate

Confidence interval: **(-9.941,11.947)**

Prediction interval: **(-21.561,23.568)**

Prediction interval extends into clinically important or unimportant effects

Heterogeneity judgment

Some concerns

Comparison AirwayScope:Miller
Evidence: indirect

NMA estimate: **5.070**

95% intervals for NMA estimate

Confidence interval: **(-5.931,16.071)**

Prediction interval: **(-17.567,27.707)**

Prediction interval extends into clinically important or unimportant effects

Heterogeneity judgment

Some concerns

Comparison AirwayScope:Truview EVO2
Evidence: indirect

NMA estimate: **-2.370**

95% intervals for NMA estimate

Confidence interval:

Prediction interval: **(-16.665,11.925)**

Confidence and **(-29.428,24.689)** *prediction intervals agree in relation to clinically important effect*

Heterogeneity judgment

No concerns

Comparison
Evidence: AirwayScope:Wis-Hipple indirect

NMA estimate: **3.870**

95% intervals for NMA estimate

Confidence interval:

Prediction interval: **(-10.234,17.974)**

Confidence and **(-22.922,30.662)** *prediction intervals agree in relation to clinically important effect*

Heterogeneity judgment

No concerns

Comparison C-MAC:Coopdech video laryngoscope
Evidence: indirect

NMA estimate: **1.152**

95% intervals for NMA estimate

Confidence interval:

Prediction interval: **(-13.052,15.355)**

Confidence and **(-25.780,28.083)** *prediction intervals agree in relation to clinically important effect*

Heterogeneity judgment

No concerns

Comparison C-MAC:GlideScope
Evidence: indirect

NMA estimate: **-10.470**

95% intervals for NMA estimate

Confidence interval: **(-24.830,3.890)**

Prediction interval: **(-37.619,16.678)**

Prediction interval extends into clinically important or unimportant effects

Heterogeneity judgment

Some concerns

Comparison C-MAC:Macintosh
Evidence: indirect

NMA estimate: **-4.970**

95% intervals for NMA estimate

Confidence interval: **(-16.054,6.114)**

Prediction interval: **(-27.714,17.773)**

Prediction interval extends into clinically important or unimportant effects

Heterogeneity judgment

Some concerns

Comparison C-MAC:McCoy
Evidence: indirect

NMA estimate: **-3.842**

95% intervals for NMA estimate

Confidence interval: **(-16.008,8.323)**

Prediction interval: **(-28.000,20.315)**

Prediction interval extends into clinically important or unimportant effects

Heterogeneity judgment

Some concerns

Comparison C-MAC:McGrath
Evidence: indirect

NMA estimate: **-4.467**

95% intervals for NMA estimate

Confidence interval: **(-17.419,8.485)**

Prediction interval: **(-29.679,20.745)**

Prediction interval extends into clinically important or unimportant effects

Heterogeneity judgment

Some concerns

Comparison C-MAC:Truview EVO2
Evidence: indirect

NMA estimate: **-7.840**

95% intervals for NMA estimate

Confidence interval: **(-20.491,4.811)**

Prediction interval: **(-32.646,16.966)**

Prediction interval extends into clinically important or unimportant effects

Heterogeneity judgment

Some concerns

Comparison Coopdech video laryngoscope:GlideScope
Evidence: indirect

NMA estimate: **-11.622**

95% intervals for NMA estimate

Confidence interval: **(-25.439,2.196)**

Prediction interval: **(-38.018,14.774)**

Prediction interval extends into clinically important or unimportant effects

Heterogeneity judgment

Some concerns

Comparison Coopdech video laryngoscope:Macintosh
Evidence: indirect

NMA estimate: **-6.122**

95% intervals for NMA estimate

Confidence interval: **(-16.493,4.250)**

Prediction interval: **(-27.962,15.718)**

Prediction interval extends into clinically important or unimportant effects

Heterogeneity judgment

Some concerns

Comparison Coopdech video laryngoscope:McCoy
Evidence: indirect

NMA estimate: **-4.994**

95% intervals for NMA estimate

Confidence interval: **(-17.779,7.791)**

Prediction interval: **(-29.980,19.992)**

Prediction interval extends into clinically important or unimportant effects

Heterogeneity judgment

Some concerns

Comparison Coopdech video laryngoscope:McGrath
Evidence: indirect

NMA estimate: **-5.619**

95% intervals for NMA estimate

Confidence interval: **(-17.967,6.730)**

Prediction interval: **(-30.019,18.782)**

Prediction interval extends into clinically important or unimportant effects

Heterogeneity judgment

Some concerns

Comparison Coopdech video laryngoscope:Miller
Evidence: indirect

NMA estimate: **-1.552**

95% intervals for NMA estimate

Confidence interval: **(-12.734,9.631)**

Prediction interval: **(-24.422,21.319)**

Prediction interval extends into clinically important or unimportant effects

Heterogeneity judgment

Some concerns

Comparison Coopdech video laryngoscope:Truview EVO2
Evidence: indirect

NMA estimate: **-8.992**

95% intervals for NMA estimate

Confidence interval: **(-23.427,5.444)**

Prediction interval: **(-36.246,18.262)**

Prediction interval extends into clinically important or unimportant effects

Heterogeneity judgment

Some concerns

Comparison Coopdech video laryngoscope:Wis-Hipple
Evidence: indirect

NMA estimate: **-2.752**

95% intervals for NMA estimate

Confidence interval:

Prediction interval: **(-16.997,11.494)**

Confidence and **(-29.741,24.238)** *prediction intervals agree in relation to clinically important effect*

Heterogeneity judgment

No concerns

Comparison GlideScope:McCoy
Evidence: indirect

NMA estimate: **6.628**

95% intervals for NMA estimate

Confidence interval: **(-5.745,19.000)**

Prediction interval: **(-17.806,31.061)**

Prediction interval extends into clinically important or unimportant effects

Heterogeneity judgment

Some concerns

Comparison GlideScope:McGrath
Evidence: indirect

NMA estimate: **6.003**

95% intervals for NMA estimate

Confidence interval: **(-5.322,17.328)**

Prediction interval: **(-17.052,29.058)**

Prediction interval extends into clinically important or unimportant effects

Heterogeneity judgment

Some concerns

Comparison GlideScope:Miller
Evidence: indirect

NMA estimate: **10.070**

95% intervals for NMA estimate

Confidence interval: **(-1.310,21.450)**

Prediction interval: **(-13.056,33.196)**

Prediction interval extends into clinically important or unimportant effects

Heterogeneity judgment

Some concerns

Comparison GlideScope:Truview EVO2
Evidence: indirect

NMA estimate: **2.630**

95% intervals for NMA estimate

Confidence interval:

Prediction interval: **(-11.959,17.219)**

Confidence and **(-24.839,30.099)** *prediction intervals agree in relation to clinically important effect*

Heterogeneity judgment

No concerns

Comparison GlideScope:Wis-Hipple
Evidence: indirect

NMA estimate: **8.870**
95% intervals for NMA estimate
Confidence interval: **(-5.531,23.272)**
Prediction interval: **(-18.337,36.077)**

Prediction interval extends into clinically important or unimportant effects

Heterogeneity judgment
Some concerns

Comparison
Evidence: Macintosh:Truview EVO2
indirect

NMA estimate: **-2.870**
95% intervals for NMA estimate
Confidence interval: **(-14.249,8.509)**
Prediction interval: **(-25.995,20.255)**

Prediction interval extends into clinically important or unimportant effects

Heterogeneity judgment
Some concerns

Comparison Macintosh:Wis-Hipple
Evidence: indirect

NMA estimate: **3.370**
95% intervals for NMA estimate
Confidence interval: **(-7.768,14.508)**
Prediction interval: **(-19.443,26.183)**

Prediction interval extends into clinically important or unimportant effects

Heterogeneity judgment
Some concerns

Comparison McCoy:McGrath
Evidence: indirect

NMA estimate: **-0.625**
95% intervals for NMA estimate
Confidence interval:
Prediction interval: **(-11.331,10.082)**

Confidence and **(-22.887,21.638)** *prediction intervals agree in relation to clinically important effect*

Heterogeneity judgment
No concerns

Comparison McCoy:Truview EVO2
Evidence: indirect

NMA estimate: **-3.998**
95% intervals for NMA estimate
Confidence interval: **(-16.433,8.438)**
Prediction interval: **(-28.514,20.519)**

Prediction interval extends into clinically important or unimportant effects

Heterogeneity judgment
Some concerns

Comparison McCoy:Wis-Hipple
Evidence: indirect

NMA estimate: **2.242**
95% intervals for NMA estimate
Confidence interval: **(-9.972,14.457)**
Prediction interval: **(-21.980,26.465)**

Prediction interval extends into clinically important or unimportant effects

Heterogeneity judgment
Some concerns

Comparison McGrath:Miller
Evidence: indirect

NMA estimate: **4.067**
95% intervals for NMA estimate
Confidence interval: **(-5.475,13.609)**
Prediction interval: **(-16.752,24.886)**

Prediction interval extends into clinically important or unimportant effects

Heterogeneity judgment
Some concerns

Comparison McGrath:Truview EVO2
Evidence: indirect

NMA estimate: **-3.373**
95% intervals for NMA estimate
Confidence interval: **(-16.579,9.832)**
Prediction interval: **(-28.930,22.184)**

Prediction interval extends into clinically important or unimportant effects

Heterogeneity judgment
Some concerns

Comparison McGrath:Wis-Hipple
Evidence: indirect

NMA estimate: **2.867**
95% intervals for NMA estimate
Confidence interval:
Prediction interval: **(-10.131,15.865)**

Confidence and **(-22.408,28.142)** *prediction intervals agree in relation to clinically important effect*

Heterogeneity judgment
No concerns

Comparison
Evidence: Truview EVO2:Wis-Hipple
indirect

NMA estimate: **6.240**
95% intervals for NMA estimate
Confidence interval: **(-6.458,18.938)**
Prediction interval: **(-18.629,31.109)**

Prediction interval extends into clinically important or unimportant effects

Heterogeneity judgment
Some concerns

Supplemental S17 Summary of findings table for the intubation time.
